# Performance of several types of beta-binomial models in comparison to standard approaches for meta-analyses with very few studies

**DOI:** 10.1186/s12874-022-01779-3

**Published:** 2022-12-13

**Authors:** Moritz Felsch, Lars Beckmann, Ralf Bender, Oliver Kuss, Guido Skipka, Tim Mathes

**Affiliations:** 1 Institute for Quality and Efficiency in Health Care (IQWiG), Cologne, Germany; 2 German Diabetes Center, Institute for Biometrics and Epidemiology, Düsseldorf, Germany; 3 Institute for Medical Statistics, University Medical Centre Göttingen, Göttingen, Germany; 4 Institute for Research in Operative Medicine, University Witten/Herdecke, Cologne, Germany

**Keywords:** Beta-binomial model, Generalised linear mixed models, Meta-analyses, Simulation study, Few studies

## Abstract

**Background:**

Meta-analyses are used to summarise the results of several studies on a specific research question. Standard methods for meta-analyses, namely inverse variance random effects models, have unfavourable properties if only very few (2 – 4) studies are available. Therefore, alternative meta-analytic methods are needed. In the case of binary data, the “common-rho” beta-binomial model has shown good results in situations with sparse data or few studies. The major concern of this model is that it ignores the fact that each treatment arm is paired with a respective control arm from the same study. Thus, the randomisation to a study arm of a specific study is disrespected, which may lead to compromised estimates of the treatment effect. Therefore, we extended this model to a version that respects randomisation.

The aim of this simulation study was to compare the “common-rho” beta-binomial model and several other beta-binomial models with standard meta-analyses models, including generalised linear mixed models and several inverse variance random effects models.

**Methods:**

We conducted a simulation study comparing beta-binomial models and various standard meta-analysis methods. The design of the simulation aimed to consider meta-analytic situations occurring in practice.

**Results:**

No method performed well in scenarios with only 2 studies in the random effects scenario. In this situation, a fixed effect model or a qualitative summary of the study results may be preferable. In scenarios with 3 or 4 studies, most methods satisfied the nominal coverage probability. The “common-rho” beta-binomial model showed the highest power under the alternative hypothesis. The beta-binomial model respecting randomisation did not improve performance.

**Conclusion:**

The “common-rho” beta-binomial appears to be a good option for meta-analyses of very few studies. As residual concerns about the consequences of disrespecting randomisation may still exist, we recommend a sensitivity analysis with a standard meta-analysis method that respects randomisation.

**Supplementary Information:**

The online version contains supplementary material available at 10.1186/s12874-022-01779-3.

## Introduction

Meta-analyses (MAs) are used to summarise the results of studies on a specific research question. If the number of studies is large and the sample sizes within these studies are not too small, standard inverse variance random effects models (IV-REMs) can provide valid estimates. However, if only a few (≤ 10) studies are included in the MA, the IV-REMs perform poorly [1–3]. The DerSimonian-Laird (DSL) method leads to too narrow 95% confidence intervals (CIs) with poor coverage probabilities below 95%, especially in the case of few studies. The Hartung-Knapp-Sidik-Jonkman (HKSJ) method generally holds the type I error, but frequently results in extremely wide 95% CIs in the case of very few (2 – 4) studies.

The worse performance in the case of few studies is a particular challenge, because such MAs are frequently performed in systematic reviews of interventions. For example, in an analysis of 14,886 MAs from the Cochrane Library, the median number of studies in MAs was 3 and the 3rd quartile was 6 [4].

In the case of binary data, alternatives to IV-REM methods have been proposed. Because outcome (success, failure) and study arm (treatment, control) for each patient can be reconstructed from studies’ four-fold tables, the generalised linear mixed model (GLMM) framework (and generally speaking all logistic regression models accounting for dependent data) can be applied to MAs [5]. The “common-rho” beta-binomial model (BBM) showed good results when pooling data of randomised controlled trials (RCTs), especially in the case of very few studies and/or rare events in the MA [6, 7]. However, there are some concerns about the model because it ignores the fact that each treatment arm is paired with a respective control arm, both originating from the same study (disrespecting the randomisation to a study arm of a specific study).

Therefore, we conducted a simulation study to compare existing BBMs and extensions with established models, such as GLMMs, HKSJ and DSL, especially in situations with very few (2 – 4) studies and for a wide range of risks, including rare events. Our focus was on BBM extensions that accounted for the pairing of a treatment arm with a control arm of the same study by implementing a random effect for the study or conditioning on the study in the maximum likelihood estimation.

The outline of the paper is as follows. In the 2nd chapter, we describe the statistical models for MAs that were included in the comparison (Models section) and explain how the simulation study was conducted (Simulation study section). In the 3rd chapter, we present the results of the simulation study. In the 4th chapter, we discuss the results, and in the 5th chapter, we conclude with final remarks and recommendations for practice.

## Methods

We consider situations where *K* studies compare a binary outcome between two study arms (*i* = 1 [or T for treatment] and *i* = 0 [or C for control]). For each study *k* (*k* = 1, …, *K*), *n*_*kT*_ and *n*_*kC*_ denote the sample size for the treatment and control arm, *y*_*kT*_ and *y*_*kC*_ the number of events in the treatment and control arm, and *θ*_*k*_ the treatment effect with a specific within-study variance ${\sigma}_k^2$. We are interested in the estimation of the overall treatment effect *θ* and use the effect measures odds ratio (OR) and relative risk (RR) to quantify the effect between the treatment and control arm.

### Models

We compared the following meta-analytic models and methods in our simulation study:

- Beta-binomial models (BBMs)
  - ◦ Standard (“common-rho”) beta-binomial model (BBST)
  - ◦ Standard beta-binomial model with an additional random treatment effect (BBFR)
  - ◦ Two “common-beta” beta-binomial models (BBCB1 and BBCB2)
- Generalised linear mixed models (GLMMs)
  - ◦ Generalised linear mixed model with a fixed intercept and random treatment effect (GLFR)
  - ◦ Generalised linear mixed model with a random intercept and random treatment effect (GLRRI)
- Inverse variance random effects model (IV-REM)
  - ◦ DerSimonian-Laird (DSL) method
  - ◦ Hartung-Knapp-Sidik-Jonkman (HKSJ) method
- Mantel-Haenszel (MH) method
- Peto odds ratio (POR) method
- Collapsed table (COLL)

and describe them in the following sections.

#### Beta-binomial models

##### Standard beta-binomial model

The standard beta-binomial model (BBST) [6, 7] assumes that the observed number of events in the control arm *y*_*kC*_ (*k* = 1, …, *K*) follows a binomial distribution *Bin*(*π*_*kC*_, *n*_*kC*_), where the event probability *π*_*kC*_ is not fixed, but beta distributed with parameters *α*_*C*_ and *β*_*C*_. These parameters are assumed to be constant over all control arms of the studies. The individual binary event *z*_*kCj*_ (*j* = 1, …, *n*_*kC*_; ${y}_{kC}=\sum_j^{n_{kC}}{z}_{kC j}$) is sampled with a different *π*_*kC*_. The expected value and variance of *π*_*kC*_ are:

$$E\left({\pi}_{kC}\right)={\mu}_C$$

and

$$Var\left({\pi}_{kC}\right)={\mu}_C\times \left(1-{\mu}_C\right)\times {\nu}_C/\left(1+{\nu}_C\right)$$

with

$${\mu}_C={\alpha}_C/\left({\alpha}_C+{\beta}_C\right)$$

and

$${\nu}_C=1/\left({\alpha}_C+{\beta}_C\right)$$

and *y*_*kC*_ is beta-binomially distributed with the expected value

$$E\left({y}_{kC}\right)={n}_{kC}\times {\mu}_C$$

and variance

$$Var\left({y}_{kC}\right)={n}_{kC}\times {\mu}_C\times \left(1-{\mu}_C\right)\times \left[1+\left({n}_{kC}-1\right)\times {\nu}_C/\left(1+{\nu}_C\right)\right].$$

Because the probabilities for two individual binary events in the control arm are sampled from the same beta distribution, these events are correlated. The intraclass correlation ${\rho}_C= corr\left({z}_{{kC j}_1},{z}_{kC{j}_2}\right)$ between two individual binary events in the control arm *k* (*k* = 1, …, *K*; *j*_1_, *j*_2_ = 1, …, *n*_*kC*_; *j*_1_ ≠ *j*_2_) is

$${\rho}_C=1/\left({\alpha}_C+{\beta}_C+1\right)$$

and is assumed to be equal over all *y*_*kC*_ (*k* = 1, …, *K*). Further, it is assumed that individual binary events from different control (and treatment) arms are uncorrelated, $corr\left({z}_{k_1{Cj}_1},{z}_{k_2C{j}_2}\right)=0$ for *k*_1_ ≠ *k*_2_.

The log likelihood of the beta-binomial distribution of all control arms can be written in closed form as

$${\ell}_C\left({\alpha}_C,{\beta}_C\right)=\sum_{k=1}^K{\ell}_{kC}\left({\alpha}_C,{\beta}_C\right)$$

with

$${\ell}_{kC}\left({\alpha}_C,{\beta}_C\right)=\log \left(\Gamma \left({n}_{kC}+1\right)\right)+\log \left(\Gamma \left({y}_{kC}+{\alpha}_C\right)\right)+\log \left(\Gamma \left({n}_{kC}-{y}_{kC}+{\beta}_C\right)\right)+\log \left(\Gamma \left({\alpha}_C+{\beta}_C\right)\right)-\log \left(\Gamma \left({y}_{kC}+1\right)\right)-\log \left(\Gamma \left({n}_{kC}-{y}_{kC}+1\right)\right)-\log \left(\Gamma \left({n}_{kC}+{\alpha}_C+{\beta}_C\right)\right)-\log \left(\Gamma \left({\alpha}_C\right)\right)-\log \left(\Gamma \left({\beta}_C\right)\right)$$

where Γ denotes the gamma function,

$${\alpha}_C={\mu}_C\times \left(1-{\rho}_C\right)/{\rho}_C$$

and

$${\beta}_C=\left(1-{\mu}_C\right)\times \left(1-{\rho}_C\right)/{\rho}_C.$$

The same formulas hold true for the number of events in the treatment arm *y*_*kT*_ (*k* = 1, …, *K*) with *n*_*kT*_, *π*_*T*_, *α*_*T*_, *β*_*T*_, *μ*_*T*_, *ν*_*T*_ and *ρ*_*T*_. The log likelihood for the treatment arms *ℓ*_*T*_(*α*_*T*_, *β*_*T*_) is given accordingly.

Importantly, in the BBST it is assumed that *ρ*_*C*_ = *ρ*_*T*_ = *ρ*, which is equivalent to *α*_*C*_ + *β*_*C*_ = *α*_*T*_ + *β*_*T*_. In other words, all individual binary events within a study arm are correlated with the same *ρ*, regardless of therapy.

The treatment effect *θ* = *b*_*T*_ = *g*^−1^(*μ*_*T*_)/*g*^−1^(*μ*_*C*_) is modelled via the link function

$$g\left({\mu}_i\right)={b}_0+{b}_T\times i$$

where *b*_0_ denotes the risk of an event in the control arm and *i* the study arm (1 = treatment; 0 = control). In our simulation study, the link functions are the logit and the natural log to measure the treatment effect as log OR and log RR.

Because *g*(*μ*_*C*_) = *b*_0_, *g*(*μ*_*T*_) = *b*_0_ + *b*_*T*_ and *α*_*C*_ + *β*_*C*_ = *α*_*T*_ + *β*_*T*_, one can write

$${b}_T=g\left({\mu}_{kT}\right)-g\left({\mu}_{kC}\right)=g\left({\alpha}_T/\left({\alpha}_T+{\beta}_T\right)\right)-g\left({\alpha}_C/\left({\alpha}_C+{\beta}_C\right)\right)=g\left({\alpha}_T/\left({\alpha}_C+{\beta}_C\right)\right)-g\left({\alpha}_C/\left({\alpha}_C+{\beta}_C\right)\right).$$

Therefore, only three parameters (*α*_*T*_, *α*_*C*_, *β*_*C*_) have to be estimated in this model.

One advantage of the BBST is that no continuity correction has to be used if there are studies with no events in one study arm (single-zero studies). Furthermore, studies without any events in both study arms (double-zero studies) are not ignored in the analysis and contribute to the overall effect estimation [6]. The only situations where the BBST cannot estimate the OR and RR (but can estimate the risk difference) are situations where no events occur in one study arm (e.g. the treatment arm) over all studies.

In the BBST, the event probability in the control arm *π*_*kC*_ is random but the treatment effect is considered to be fixed across all studies. Thus, although the BBST is a true random effects model, from a meta-analytic point of view, it is a model with a fixed treatment effect.

Furthermore, the BBST estimates the treatment effect via *μ*_*T*_ and *μ*_*C*_. Therefore, the fact that the treatment and control arm originate from the same study is ignored in the process of parameter estimation. Thus, the BBST disrespects the randomisation to a study arm of a specific study. According to Senn [8] and Piepho et al. [9] it is unlikely that this property is detrimental in situations where the same treatments are evaluated across trials, because the effects are comparable between the studies. This was indicated by recent simulation results, where the BBST performed well [6, 7].

##### Standard beta-binomial model with additional random effect

To deal with the aforementioned properties of BBST as a fixed effect model that disrespects randomisation, we implemented another BBM (BBFR) where the treatment effect *θ* = *b*_*T*_ = *g*^−1^(*μ*_*T*_)/*g*^−1^(*μ*_*C*_) is modelled as

$$g\left({\mu}_i\right)={b}_0+\left({b}_T+{\gamma}_k\right)\times i$$

with ${\gamma}_k\sim N\left(0,{\sigma}_{BBFR}^2\right)$. By adding a random effect to the treatment effect, this model respects the randomisation to a study arm of a specific study.

Like the BBST, the BBFR takes the information of all studies into account and therefore needs no continuity correction when single- or double-zero studies are included in the MA.

When constructing the 95% CI for the treatment effect *b*_*T*_, Mathes and Kuss [7] showed that using the t-distribution rather than the normal distribution led to better performance of the BBST. Therefore, for both models (BBST, BBFR) we calculated 95% CIs for the treatment effect *b*_*T*_ using the t-distribution

$${\hat{b}}_{T(BB)}\pm {t}_{df;0.975}\times {\hat{\sigma}}_{BB},$$

where ${\hat{b}}_{T(BB)}$ is the estimated treatment effect of BBST or BBFR, ${\hat{\sigma}}_{BB}$ the corresponding estimated standard error and *t*_*df*; 0.975_ the 0.975 quantile of the t-distribution with *df* degrees of freedom. We chose two different numbers of degrees of freedom: *df* = *K* − 1, which are the degrees of freedom for the HKSJ method, and *df* = 2*K* − 2, which is a reasonable compromise between the 2*K* and 2*K* − 3 degrees of freedom that were used by Mathes and Kuss [7] and that led to too narrow and too wide intervals, respectively.

##### Common-beta BBM

In the BBST it is assumed that the intraclass correlation *ρ* is equal for all treatment and control arms implying that *α*_*C*_ + *β*_*C*_ = *α*_*T*_ + *β*_*T*_ holds true. Therefore, this model is sometimes called the “common-rho” model. Another possibility is to assume that *β* is equal in all groups (*β*_*C*_ = *β*_*T*_ = *β*). We call this model the “common-beta” BBM. Similar to the BBST, the likelihood function of the “common-beta” BBM can be written in closed form. Guimaraes [10], and more recently in the meta-analytic context Mathes and Kuss [11], were able to show that a negative binomial regression model for count panel data can be interpreted as this “common-beta” BBM.

We considered two versions of the “common-beta” BBM in our simulation. BBCB1, which disrespects the randomisation to a study arm of a specific study by conditioning on study group, while BBCB2 respects the randomisation to a study arm of a specific study by conditioning on the study.

As for BBST and BBFR, we constructed 95% CIs using the t-distribution for the treatment effect *b*_*T*_

$${\hat{b}}_{T(BBCB)}\pm {t}_{df;0.975}\times {\hat{\sigma}}_{BBCB},$$

where ${\hat{b}}_{T(BBCB)}$ is the estimated treatment effect of BBCB1 or BBCB2, ${\hat{\sigma}}_{BBCB}$ the corresponding estimated standard error, and *t*_*df*; 0.975_ the 0.975 quantile of the t-distribution with *df* = *K* − 1 or 2*K* − 2 degrees of freedom.

#### Generalised linear mixed models

Generalised linear mixed models (GLMMs) [5] are probably the most common alternatives to standard MA methods (Inverse variance random effects models section) with binary data because of their flexibility. A GLMM with random treatment effect *θ*_*k*_ = *θ* + *ϵ*_*k*_ can be expressed as

$$g\left({\pi}_{ki}\right)={\gamma}_k+i\times \theta +i\times {\epsilon}_k$$

where *g*(∙) is the link function for the OR (logit) or RR (log), *π*_*ki*_ the probability of an event in study arm *i* (*i* = 1: treatment; *i* = 0: control) of study *k* (*k* = 1, …, *K*), *γ*_*k*_ the intercept (baseline risk of an event) of study *k* and *ϵ*_*k*_~*N*(0, *τ*^2^).

We included two GLMMs in our simulation. The first model has a fixed intercept and a random treatment effect (GLFR) (similar to model 2 in Jackson et al. [5] originally suggested by Simmonds and Higgins [12]). The second GLMM included has a random intercept (${\gamma}_k\sim N\left(\gamma, {\sigma}_{GLRRI}^2\right)$) and an uncorrelated random treatment effect (GLRRI), similar to model 3 in Jackson et al. [5].

Like the BBST, the GLMM is a random effects model. But as the treatment effect is random it is more comparable to meta-analytic REMs than the BBST.

Like BBMs, GLMMs take the information of all studies into account and therefore do not need a continuity correction for single- or double-zero studies.

We calculated 95% CIs for log OR and log RR using normal approximation, therefore

$${\hat{\theta}}_{GLMM}\pm 1.96\times {\hat{\sigma}}_{GLMM},$$

where ${\hat{\theta}}_{GLMM}$ is the estimated overall effect (log OR or log RR) in the analysed model (GLFR or GLRRI) and ${\hat{\sigma}}_{GLMM}$ the corresponding standard error.

#### Inverse variance random effects models

The meta-analytic random effects model (REM) assumes no common effect for all studies but instead assumes that the mean of all study effects is the mean of the distribution of the true effect [13]. The study effects are usually assumed to follow a normal distribution. The treatment effect in study *k* can be expressed as ${\hat{\theta}}_k={\theta}_k+{\varepsilon}_k$ with *θ*_*k*_ = *θ*_*REM*_ + *δ*_*k*_, *δ*_*k*_~*N*(0, *τ*^2^) and ${\varepsilon}_k\sim N\left(0,{\sigma}_k^2\right)$.

The overall effect *θ*_*REM*_ of this REM can be estimated by the inverse variance approach

$${\hat{\theta}}_{REM}=\frac{\sum_{k=1}^K{w}_{k(REM)}\times {\hat{\theta}}_k}{\sum_{k=1}^K{w}_{k(REM)}},$$

where ${w}_{k(REM)}=1/\left({\sigma}_k^2+{\tau}^2\right)$ are the study-specific weights, ${\sigma}_k^2$ is the within-study variance, and *τ*^2^ is the between-study variance (heterogeneity).

In the case of binary data and for OR and RR as the effect sizes, ${\hat{\theta}}_k$ and ${\hat{\theta}}_{REM}$ are generally analysed on the logarithmic scale, thus representing the log OR and the log RR. A continuity correction is applied to single-zero studies by adding 0.5 to the number of events in both groups. Double-zero studies are ignored for parameter estimation.

##### DerSimonian-Laird method

The DerSimonian-Laird (DSL) method [14] was regarded as the gold standard for performing MAs until it was criticized in recent years for being anti-conservative (i.e., producing too narrow CIs) [15].

The DSL estimator ${\hat{\theta}}_{DSL}$ uses weights ${w}_{k(DSL)}=1/\left({\sigma}_k^2+{\tau}_{DSL}^2\right)$ where

$${\tau}_{DSL}^2=\max \left\{0,\frac{Q-\left(K-1\right)}{\sum_{k=1}^K{w}_{k(FEM)}-\frac{\sum_{k=1}^K{w}_{k(FEM)}^2}{\sum_{k=1}^K{w}_{k(FEM)}}}\right\}$$

is estimated using the method-of-moments principle [14, 16]. Q is the heterogeneity statistic $Q=\sum_{k=1}^K{w}_{k(FEM)}\times {\left({\hat{\theta}}_k-{\hat{\theta}}_{FEM}\right)}^2$ and ${\hat{\theta}}_{FEM}$ is the pooled effect of a fixed effect model with weights ${w}_{k(FEM)}=1/{\sigma}_k^2$.

The 95% CI for *θ*_*REM*_ is given by

$${\hat{\theta}}_{DSL}\pm 1.96\times {\hat{\sigma}}_{DSL}.$$

The standard error is given by

$${\hat{\sigma}}_{DSL}=\sqrt{1/\left(\sum_{k=1}^K{w}_{k(DSL)}\right)}.$$

We included the DSL method in our simulation because it is still in use and is important, at least for sensitivity analyses [17].

##### Hartung-Knapp-Sidik-Jonkman method using the Paule-Mandel heterogeneity variance estimator

For the method of Hartung-Knapp-Sidik-Jonkman (HKSJ) [18, 19] different weights *w*_*k*(*HKSJ*)_ can be applied to calculate the overall estimator ${\hat{\theta}}_{HKSJ}$, depending on what estimator for the between-study variance (heterogeneity) is used. For the analysis presented here, we used ${w}_{k(HKSJ)}={w}_{k(PM)}=1/\left({\sigma}_k^2+{\tau}_{PM}^2\right)$, where ${\tau}_{PM}^2$ is the Paule-Mandel (PM) estimator of *τ*^2^ [20–22]. The PM estimator of *τ*^2^ is derived from the generalised Q statistic

$$Q\left({\tau}_{PM}^2\right)=\sum_{k=1}^K{w}_{k(PM)}\times {\left({\hat{\theta}}_k-\hat{\theta}\left({\tau}_{PM}^2\right)\right)}^2$$

by setting $Q\left({\tau}_{PM}^2\right)$ to its expected value *K* − 1 with ${w}_{k(PM)}=1/\left({\sigma}_k^2+{\tau}_{PM}^2\right)$ and $\hat{\theta}\left({\tau}_{PM}^2\right)=\left(\sum_{k=1}^K{w}_{k(PM)}\times {\hat{\theta}}_k\right)/\left(\sum_{k=1}^K{w}_{k(PM)}\right)$. The equation is solved by iterating ${\tau}_{PM}^2$ until convergence is reached. If no solution exists, ${\hat{\tau}}_{PM}^2$ is set to 0.

The 95% CI for *θ*_*REM*_ is given by

$${\hat{\theta}}_{HKSJ}\pm {t}_{K-1;0.975}\times \sqrt{q}\times {\hat{\sigma}}_{HKSJ}$$

where *t*_*K* − 1; 0.975_ is the 0.975 quantile of the t-distribution with *K* − 1 degrees of freedom,

$$q=\frac{1}{K-1}\sum_{k=1}^K{w}_{k(HKSJ)}\times {\left({\hat{\theta}}_k-{\hat{\theta}}_{HKSJ}\right)}^2$$

and

$${\hat{\sigma}}_{HKSJ}=\sqrt{1/\left(\sum_{k=1}^K{w}_{k(HKSJ)}\right)}.$$

In general, this CI tends to be wider than the 95% CI of the DSL method. However, in very homogeneous data situations, this is not always the case. Therefore, Knapp and Hartung [23] suggested an ad hoc modification of *q*, *q*^∗^ = max {1, *q*}. If the ad hoc modification is used, the 95% CI of the HKSJ method is always wider than the 95% CI of the DSL method. In the simulation study, we followed the recommendations of the literature to carry out sensitivity analyses using a fixed effect model or the DSL method to decide on whether the modification is needed [17, 24]. If the 95% CI of HKSJ was narrower than the 95% CI of DSL, the ad hoc modification was used.

We included the HKSJ method in our simulation because it is well-established that it performs better than the DSL method [17] and is recommended as the standard approach in the Cochrane Handbook in combination with the PM estimator for *τ*^2^ [25]. Furthermore, it is the IQWiG standard method for MAs with five or more studies [26]. It is well-known that for MAs with less than five studies, 95% CIs of the HKSJ method tend to be too wide but in general, the method does not lead to increased type I errors.

#### Other models

##### Mantel-Haenszel method

The Mantel-Haenszel (MH) method [27] assumes a fixed effect model which is based on the assumption that all studies in the MA have a common effect *θ*_*FEM*_. The resulting estimate is a weighted average of the study-specific risk ratios or risk differences.

The MH odds ratio of the overall effect is given by

$${OR}_{MH}=\frac{\sum_{k=1}^K{w}_{k(MH)}\times {\hat{OR}}_{k(MH)}}{\sum_{k=1}^K{w}_{k(MH)}}$$

where ${\hat{OR}}_{k(MH)}=\frac{y_{kT}\times \left({n}_{kC}-{y}_{kC}\right)}{\left({n}_{kT}-{y}_{kT}\right)\times {y}_{kC}}$ is the estimated odds ratio, ${w}_{k(MH)}=\frac{\left({n}_{kT}-{y}_{kT}\right)\times {y}_{kC}}{n_k}$ the weight, and *n*_*k*_ = *n*_*kT*_ + *n*_*kC*_ the sample size of study *k* (*k* = 1, …, *K*).

No continuity correction was applied. Therefore, single- and double-zero studies were ignored during the analysis. We estimated 95% CIs using normal approximation.

We included the MH method in our analysis because it is the standard fixed effect model for binary data in Cochrane Reviews and performs better than the standard (inverse variance) fixed effect model in the case of sparse data [25].

##### Peto odds ratio method

The Peto odds ratio (POR) method [28] was introduced as the effect estimator for the real underlying OR in the data situation of rare events. Later it was shown that POR is an independent effect measure and cannot be used as approximation of the true OR in all (rare event) data situations [29].

The pooled log POR is estimated as

$$\log (POR)=\frac{\sum_{k=1}^K\left({O}_k-{E}_k\right)}{\sum_{k=1}^K{V}_k},$$

where *O*_*k*_ is the observed number of events in the treatment arm, *E*_*k*_ the expected number of events in the treatment arm under the assumption of no treatment effect, and *V*_*k*_ the variance of their difference.

No continuity correction was applied. Single-zero studies are included in the analysis by definition, whereas double-zero studies are ignored. We estimated 95% CIs using the normal approximation.

Although this method has major limitations [29, 30], we considered the POR in our analysis because according to the Cochrane Handbook, it is the standard MA method for small intervention effects or very rare events [25].

##### Collapsed table

This method (COLL) ignores the fact that data were collected from different studies and aggregates them in one single four-fold table. The effect is estimated using standard methods for 2 × 2 tables [31]. The procedure assumes a constant underlying risk of an event across all studies, which is rarely given, and therefore the method is vulnerable to Simpson’s paradox [32, 33].

Because of its simplicity and because the differences in event rates across studies might be negligible in scenarios with few events and studies, we included the method in our analysis.

We applied a continuity correction if necessary and estimated 95% CIs using normal approximation.

### Simulation study

We performed a simulation study using SAS/STAT® software Version 9.4 (SAS Institute Inc., Cary, NC, USA) for Microsoft Windows 10 to compare the statistical properties of the different meta-analytic methods. In an attempt to investigate the methods in realistic scenarios, the values of the design factors in the simulation study were chosen from MAs actually performed. For this purpose, we used the review by Turner et al. [4] The review included 1991 systematic reviews from the Cochrane Database of Systematic Reviews and analysed 14,886 MAs of dichotomous outcomes from 77,237 single studies.

#### Design of simulations

In our simulation study, we varied the following parameters: the number of studies in the MA, the sample size of a single study, the event probability in the control arm, the heterogeneity between studies in the MA, and the effect size in the MA.

We chose 2, 3, 4, 5 and 10 as the number of studies in the MA. Ten studies were chosen in addition to gain an impression of how the models perform in rather uncritical scenarios. For each number of studies in the MA, we simulated 10,000 data sets each under the null (H_0_) and under the alternative hypothesis (H_1_). In each data set, we sampled the values of the other parameters from distributional functions based on the data from Turner et al. [4] For example, the sample size *n*_*k*_ for a single study in the MA was sampled using a log-normal distribution with *μ*_*ND*_ = 4.615 and *σ*_*ND*_ = 1.1, resulting in a mean overall sample size of 185.7 (median: 102.0; 1st quartile: 50.0; 3rd quartile: 213.0). Table 1 shows the distributional assumptions and the resulting data values. The data were simulated according to the REM from the Inverse variance random effects models section. The simulation process was as follows:

1. For each MA, we sampled the true risk *π*_*C*, *true*_ in the control arm and the heterogeneity *τ*^2^ between the studies in the MA from the distributional functions given in Table 1. Under H_1_, we did the same for the effect size *θ* = log OR and log RR. Under H_0_, the effect size was set to zero (i.e. OR = 1 and RR = 1).
2. For the *k*th study in the MA, we
  - sampled the study size *n*_*k*_ and the size of the treatment (*n*_*kT*_) and control arm (*n*_*kC*_) using the distributional functions given in Table 1
  - sampled the number of events in the control arm *y*_*kC*_ using a binomial distribution with *π*_*C*, *true*_ as event probability and *n*_*kC*_ as number of experiments
  - sampled an individual heterogeneity variance ${\tau}_k^2$ using the sampled true heterogeneity and assuming that it follows a normal distribution within the *k*th MA
  - calculated the true risk in the treatment arm *π*_*kT*, *true*_ using *π*_*C*, *true*_, *θ*, ${\tau}_k^2$ and the following formula: ${\pi}_{kT, true}=\exp \left(\textrm{logit}\left({\pi}_{C, true}\right)+\theta +{\tau}_k^2\right)/\left(1+\exp \left(\textrm{logit}\left({\pi}_{C, true}\right)+\theta +{\tau}_k^2\right)\right)$
  - sampled the number of events in the treatment arm *y*_*kT*_ using a binomial distribution with *π*_*kT*, *true*_ as event probability and *n*_*kT*_ as number of experiments.

**Table 1:** Description of the simulation study

Parameter	Distributional assumption and parameter specification	Description of resulting data set
Sample size of single study *n*_*k*_	Generated from a log-normal distribution with *μ*_*ND*_ = 4.615 and *σ*_*ND*_ = 1.1	Q1 = 50.0, median = 102.0, mean = 185.7, Q3 = 213.0
Sample size of treatment (control) arm of single study *n*_*kT*_ (*n*_*kC*_)	For *n*_*kT*_: Generated from a binomial distribution with event probability 0.5 (1:1 randomisation) and *n*_*k*_ as number of experiments For *n*_*kC*_: *n*_*k*_ − *n*_*kT*_	
Event probability in control group *π*_*C*, *true*_	Generated from a beta distribution with *α* = 0.42 and *β* = 1.43	Q1 = 0.024, median = 0.129, mean = 0.230, Q3 = 0.369
Variation *σ*^2^ within study	Is implicitly given by random sample size of single study and event probability in control group	
Heterogeneity *τ*^2^ between the studies (for *θ* = log OR)	Generated from a log normal distribution with *μ*_*ND*_ = −1.47, *σ*_*ND*_ = 1.65 and skewness = − 0.55 using Fleishman’s power transformation to generate the skewed distribution [34, 35]	*τ*^2^: Q1 = 0.079, median = 0.273, mean = 0.621, Q3 = 0.802
Effect size of *θ* = log OR under H_1_	Generated from a log normal distribution with *μ*_*ND*_ = − 0.59, *σ*_*ND*_ = 0.61, skewness = − 1.28 and kurtosis = 3.68 using Fleishman’s power transformation [34, 35]	OR: Q1 = 0.527, mean = 0.673, median = 0.694, Q3 = 0.838

*OR* odds ratio, *SD* standard deviation, Q1: 1st quartile; Q3: 3rd quartile

Overall, we simulated 5 (number of studies in the MA 2, 3, 4, 5, 10) × 2 (under H_0_ and under H_1_) × 10,000 data sets = 100,000 MAs each for the OR and for the RR.

We performed a sensitivity analysis to assess the robustness of the results regarding heterogeneity. For each MA, we calculated Cochran’s Q test [13] in order to gain an impression of whether the results depend on homogeneity of the data situations. Although we are aware that this dichotomisation is somewhat arbitrary, we used the Cochran’s Q test for the purpose of sensitivity analyses because in practical applications, MAs will frequently not be performed, at least when the test for heterogeneity is statistically significant.

#### Parameter estimation in the models

For parameter estimation, we used the SAS/STAT® software procedure NLMIXED for BBST and BBFR, COUNTREG for BBCB1 and BBCB2, GLIMMIX for GLFR and GLRRI, and FREQ for MH and COLL. For HKSJ, DSL and POR, we programmed our own syntax that was validated using R 3.3 [36] and the metafor package [37].

Because we used the COUNTREG procedure for parameter estimation, we were only able to estimate the OR but not the RR. In the GLIMMIX procedure, we used maximum likelihood estimation based on adaptive quadrature (METHOD = QUAD) with 1 quadrature point (QPOINTS = 1), which is equivalent to the Laplace approximation. We decided to use the Laplace approximation because we assumed that this would be most robust [38].

#### Performance measures

To assess the performance of the methods we calculated the following measures:

- Number of converged simulation runs with estimated effect and standard error (*R*): Sometimes the procedures converged and an effect was estimated but no standard error was given (most notably when using the NLMIXED procedure). Because such results would cause problems of interpretation, we counted these runs as non-converged. All other measures were based on *R*, the number of converged simulation runs with an estimated effect and standard error.
- (Absolute) bias ${\hat{\theta}}_r-{\theta}_r$: Difference between the estimated (${\hat{\theta}}_r$) and true effect (*θ*_*r*_); *r* = 1, …, *R*.
- Percentage bias under H_1_$\left(100\times \left({\hat{\theta}}_r-{\theta}_r\right)\right)/{\theta}_r$: Ratio of the bias (${\hat{\theta}}_r-{\theta}_r$) and true effect (*θ*_*r*_); *r* = 1, …, *R*.
- Coverage probability: Proportion of converged simulation runs where the 95% CI included the true effect *θ*_*r*_; *r* = 1, …, *R*.
- Length of 95% CI: Difference (*CI*_*U*, *r*_ − *CI*_*L*, *r*_) of upper (*CI*_*U*, *r*_) and lower (*CI*_*L*, *r*_) confidence limit of the 95% CI for *θ*_*r*_; *r* = 1, …, *R*.
- Power under H_1_: Proportion of converged simulation runs under H_1_ where the 95% CI excluded the null effect.

Bias, percentage bias and length of 95% CI were calculated on the corresponding log scale, i.e. log OR or log RR. For these measures, the median as well as the 1st and 3rd quartile are presented.

The simulation code containing the data generation, parameter estimation, and the calculation of the performances measures is available in the Supporting Information.

## Results

In the following sections, we describe the results for the OR of all methods under the null hypothesis (Results for the odds ratio under the null hypothesis section), and alternative hypothesis (Results for the odds ratio under the alternative hypothesis section). In the  Direct comparison of results for beta-binomial models section, we compare the results of the BBMs, especially BBST and BBFR. The results of the RR are discussed in the Results for the relative risk section. The results of the sensitivity analysis are presented in the Sensitivity analysis section and the main results are summarized in the Summary of results section.

Although the results of all methods are described in this chapter, in tables and figures we focused on the BBST, BBCB1, BBCB2, GLFR, HKSJ and DSL. As BBST and BBFR yielded almost identical results (Direct comparison of results for beta-binomial models section and Discussion), we refrained from showing them both in tables and figures. The results of all methods can be found in the Supporting Information.

For the BBMs, the results for coverage probability, length of 95% CI and power, with CIs using 2*K* − 2 degrees of freedom, are presented and discussed. Results for the CIs with *K* − 1 degrees of freedom, which were generally worse, can be found in the Supporting Information.

### Results for the odds ratio under the null hypothesis

#### Number of converged simulation runs

**Fig. 1:**
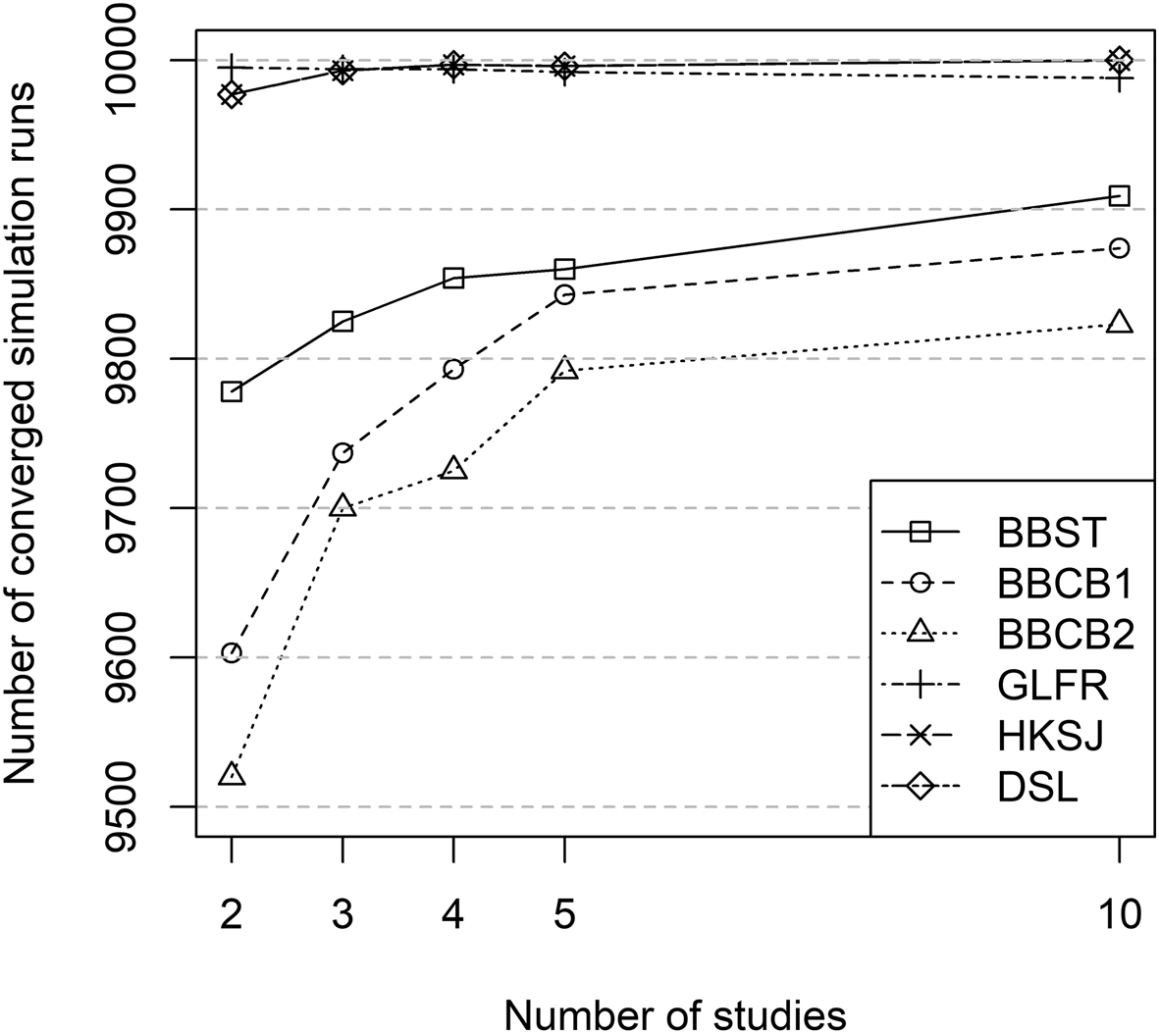
Number of converged simulation runs (out of 10,000) for the OR under the null hypothesis

The methods HKSJ, DSL, COLL and POR were only marginally affected by convergence problems (< 0.5% for all scenarios) due to their construction. The same held true for GLFR (*R* ≥ 9988 for all scenarios). The BBMs (BBST, BBFR, BBCB1, BBCB2) converged in more than 95% of the simulation runs. The number of converged runs was lower for MH in scenarios with 2 studies (*R* = 9394) but increased up to 10,000 in scenarios with 10 studies. The GLRRI had the lowest number of converged runs, with 8469 (2 studies) to 6896 runs (10 studies) (see Fig. 1 and Table S1 in the Supporting Information).

#### Bias

For all methods, the median bias was similar, mainly positive, increased with an increasing number of studies, and was low (< 0.04 on log OR scale). Because bias was calculated on the log OR scale (bias $=\log \left(\hat{OR}\right)-\log (OR)=\log \left(\hat{OR}/ OR\right)$), this could be interpreted as relative effect of the ORs. Therefore, the estimated OR increased up to 4% in median if compared to the true OR (see Table 2 and Table S2 in the Supporting Information).

**Table 2:** (Absolute) bias for the log OR under the null hypothesis

Number of studies	BBST	BBCB1	BBCB2	GLFR	HKSJ	DSL
	Median	Median	Median	Median	Median	Median
	Q1	Q1	Q1	Q1	Q1	Q1
	Q3	Q3	Q3	Q3	Q3	Q3
2	0.0004	−0.0090	− 0.0000	0.0112	0.0065	0.0065
	−0.3857	−0.3957	−0.2880	−0.3846	−0.3692	−0.3692
	0.3907	0.3652	0.2877	0.4325	0.3880	0.3880
3	0.0109	−0.0098	−0.0000	0.0239	0.0184	0.0186
	−0.2994	−0.3262	−0.2476	−0.2965	−0.2783	−0.2765
	0.3497	0.3253	0.2515	0.3963	0.3405	0.3388
4	0.0119	−0.0058	− 0.0000	0.0229	0.0144	0.0148
	−0.2522	− 0.2772	−0.2151	− 0.2466	−0.2346	−0.2335
	0.2943	0.2657	0.2179	0.3326	0.2905	0.2890
5	0.0167	−0.0074	0.0000	0.0250	0.0196	0.0200
	− 0.2099	− 0.2468	− 0.1815	− 0.2097	−0.2049	−0.2022
	0.2640	0.2345	0.1894	0.3066	0.2654	0.2666
10	0.0176	−0.0051	0.0000	0.0293	0.0221	0.0230
	−0.1438	−0.1815	−0.1293	−0.1336	−0.1297	−0.1293
	0.1883	0.1571	0.1336	0.2248	0.1935	0.1934

Q1: 1st quartile; Q3: 3rd quartile

#### Coverage probability

**Fig. 2:**
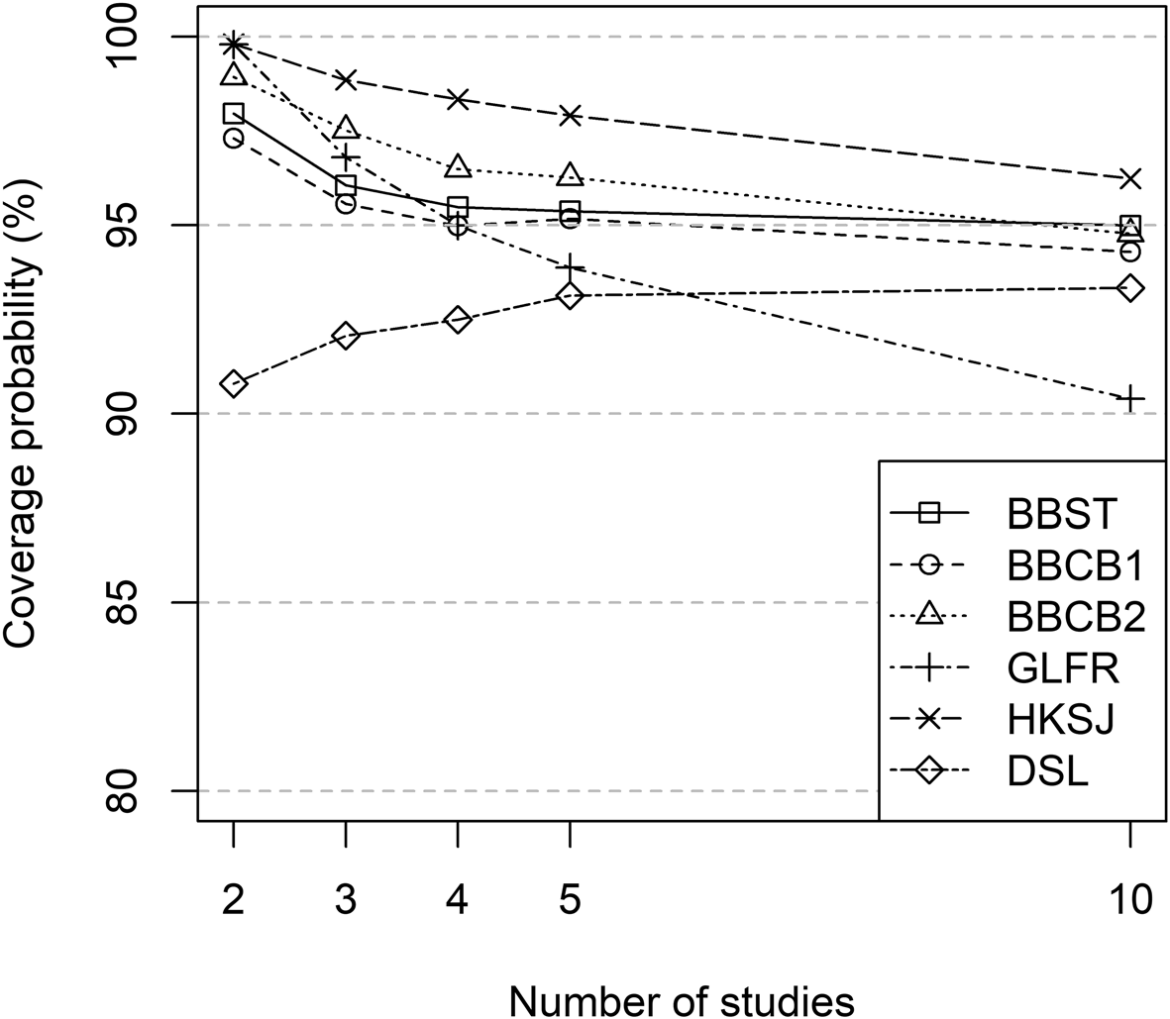
Coverage probability (%) for the OR under the null hypothesis (95% CI with 2K − 2 degrees of freedom for BBST, BBCB1 and BBCB2)

Coverage probabilities were at or above 95% for the BBMs (BBST, BBFR, BBCB1, BBCB2) and HKSJ for all scenarios. Coverage probability for GLFR was at or above 95% for 2, 3 and 4 studies and fell below 95% for 5 (93.9%) and 10 studies (90.4%). All other methods had coverage probabilities below 95% for all scenarios (GLRRI: ≤ 85.7%; DSL: ≤ 93.3%; MH: ≤ 81.6%; POR: ≤ 80.6%; COLL: ≤ 82.2%) (see Fig. 2 and Table S3 in the Supporting Information).

BBMs (BBST, BBFR, BBCB1, BBCB2), HKSJ and GLFR had coverage probabilities ≥97.3% in scenarios with 2 studies. In scenarios with 3, 4, 5, and 10 studies, BBMs (BBST, BBFR, BBCB1, BBCB2) were closer to 95% than HKSJ (≥ 96.2% in all scenarios). The same applied to the GLFR in scenarios with 3 and 4 studies (see Fig. 2 and Table S3 in the Supporting Information).

Coverage probabilities of the BBMs were closer to 95%, i.e., less conservative, if confidence intervals with 2*K* − 2 degrees of freedom were used compared to the use of K – 1 degrees of freedom (see Table S3 in the Supporting Information).

#### Length of 95% CI

The length of the 95% CI for log OR was largest and approximately the same for HKSJ and GLFR in the scenario with 2 studies. The length was far shorter for BBMs. With an increasing number of studies in the MA, the length of the 95% CIs converged between the methods, but HKSJ always had the widest CIs (see Table 3 and Table S4 in the Supporting Information).

**Table 3:** Length of 95% CI for the log OR under the null hypothesis (95% CI with 2K − 2 degrees of freedom for BBST, BBCB1 and BBCB2)

Number of studies	BBST	BBCB1	BBCB2	GLFR	HKSJ	DSL
	Median	Median	Median	Median	Median	Median
	Q1	Q1	Q1	Q1	Q1	Q1
	Q3	Q3	Q3	Q3	Q3	Q3
2	4.4403	4.3872	3.6847	12.4947	14.1601	2.5465
	2.6654	2.6242	2.0261	7.5389	7.7049	1.4191
	7.9314	7.7574	6.9888	23.3022	25.3066	4.2315
3	2.4496	2.4603	2.0577	3.4106	4.3827	2.0281
	1.4494	1.4500	1.0987	2.0936	2.5054	1.1865
	4.2823	4.2821	3.7885	6.3203	6.8549	3.2792
4	1.8850	1.9022	1.5594	2.1652	2.8089	1.7153
	1.1225	1.1241	0.8478	1.3430	1.6347	1.0198
	3.1821	3.2001	2.8554	3.8901	4.3356	2.7188
5	1.6165	1.6338	1.3294	1.6974	2.1806	1.5158
	0.9599	0.9652	0.7283	1.0538	1.2838	0.9046
	2.6942	2.7002	2.3815	3.0376	3.3875	2.3956
10	1.0760	1.0796	0.8550	0.9788	1.2340	1.0463
	0.6320	0.6351	0.4707	0.6012	0.7326	0.6255
	1.7404	1.7336	1.5407	1.7447	1.9143	1.6381

Q1: 1st quartile; Q3: 3rd quartile

### Results for the odds ratio under the alternative hypothesis

The results under the alternative hypothesis were quite similar to the results under the null hypothesis for all performance measures investigated. Therefore, for the number of converged simulation runs, bias, squared error, coverage probability and length of 95% CI, only important differences between null and alternative hypothesis are mentioned.

#### Number of converged simulation runs

The number of converged runs for GLRRI increased by about 100 runs up to between 8590 (2 studies) and 7083 runs (10 studies). GLRRI still had the lowest convergence rate (see Table S5 in the Supporting Information).

#### Bias

The median absolute bias of log OR was higher than under the null hypothesis and mainly positive, but still small (< 0.05), for most methods. The most notable exception was BBCB2 with a median bias > 0.10 for all scenarios (see Table 4 and Table S6 in the Supporting Information).

**Table 4:** (Absolute) bias for the log OR under the alternative hypothesis

Number of studies	BBST	BBCB1	BBCB2	GLFR	HKSJ	DSL
	Median	Median	Median	Median	Median	Median
	Q1	Q1	Q1	Q1	Q1	Q1
	Q3	Q3	Q3	Q3	Q3	Q3
2	0.0203	0.0224	0.1378	0.0251	0.0478	0.0478
	−0.4137	−0.3925	−0.2316	−0.4195	−0.3418	−0.3418
	0.4385	0.4287	0.4747	0.4758	0.4699	0.4699
3	0.0225	0.0131	0.1327	0.0283	0.0608	0.0620
	−0.3057	−0.3130	−0.1817	−0.3117	−0.2443	−0.2392
	0.3762	0.3609	0.4300	0.3990	0.4080	0.4067
4	0.0215	0.0081	0.1258	0.0220	0.0545	0.0553
	−0.2610	−0.2798	− 0.1505	− 0.2676	− 0.2060	− 0.2029
	0.3136	0.2917	0.3761	0.3335	0.3499	0.3516
5	0.0209	0.0065	0.1187	0.0227	0.0535	0.0545
	−0.2298	−0.2563	−0.1278	−0.2384	−0.1639	−0.1634
	0.2830	0.2595	0.3606	0.3057	0.3232	0.3252
10	0.0207	0.0011	0.1073	0.0235	0.0525	0.0532
	−0.1409	−0.1684	−0.0698	−0.1486	−0.0984	−0.0984
	0.2119	0.1779	0.2858	0.2244	0.2556	0.2582

Q1: 1st quartile; Q3: 3rd quartile

#### Percentage bias

The percentage bias of log OR was defined as $\left(100\times \left(\log \left(\hat{OR}\right)-\log (OR)\right)\right)/\log (OR)$ and because log OR < 0, a negative percentage bias means an overestimation of the log OR. The median percentage bias was low (about − 6 and 0%) for the BBMs (BBST, BBFR, BBCB1), except for BBCB2, and the GLMMs (GLFR, GLRRI). The median values for HKSJ, DSL, MH, POR and COLL were higher, with values between − 14 and − 7%. BBCB2 had much worse median values, about − 30% (see Table 5 and Table S7 in the Supporting Information).

**Table 5:** Percentage bias (%) for the log OR under the alternative hypothesis

Number of studies	BBST	BBCB1	BBCB2	GLFR	HKSJ	DSL
	Median	Median	Median	Median	Median	Median
	Q1	Q1	Q1	Q1	Q1	Q1
	Q3	Q3	Q3	Q3	Q3	Q3
2	−5.11	−5.19	−36.47	−6.08	−11.18	−11.18
	− 123.48	− 121.31	−108.22	− 134.08	−129.21	−129.21
	129.70	120.92	71.37	131.63	95.25	95.25
3	−5.15	−3.16	−35.18	−6.79	−14.07	−13.90
	−109.29	− 104.89	−100.79	− 120.90	− 113.60	−113.90
	88.72	91.03	53.83	90.55	67.96	66.87
4	−4.71	−1.71	−32.30	−5.23	−12.62	−12.57
	−88.62	−82.00	−99.91	−96.81	−95.54	−96.22
	77.02	82.45	47.71	78.32	57.25	56.93
5	−5.07	−1.44	−31.51	− 5.24	−12.35	−12.74
	−81.65	−72.16	−91.35	−91.15	−89.98	−90.59
	65.71	74.12	38.30	66.97	47.04	45.97
10	−5.11	−0.26	−28.08	−5.71	−12.83	−12.84
	−55.87	−47.14	−71.48	−62.81	−66.42	−66.33
	40.20	48.89	20.74	41.06	27.47	27.21

Q1: 1st quartile; Q3: 3rd quartile

#### Coverage probability

**Fig. 3:**
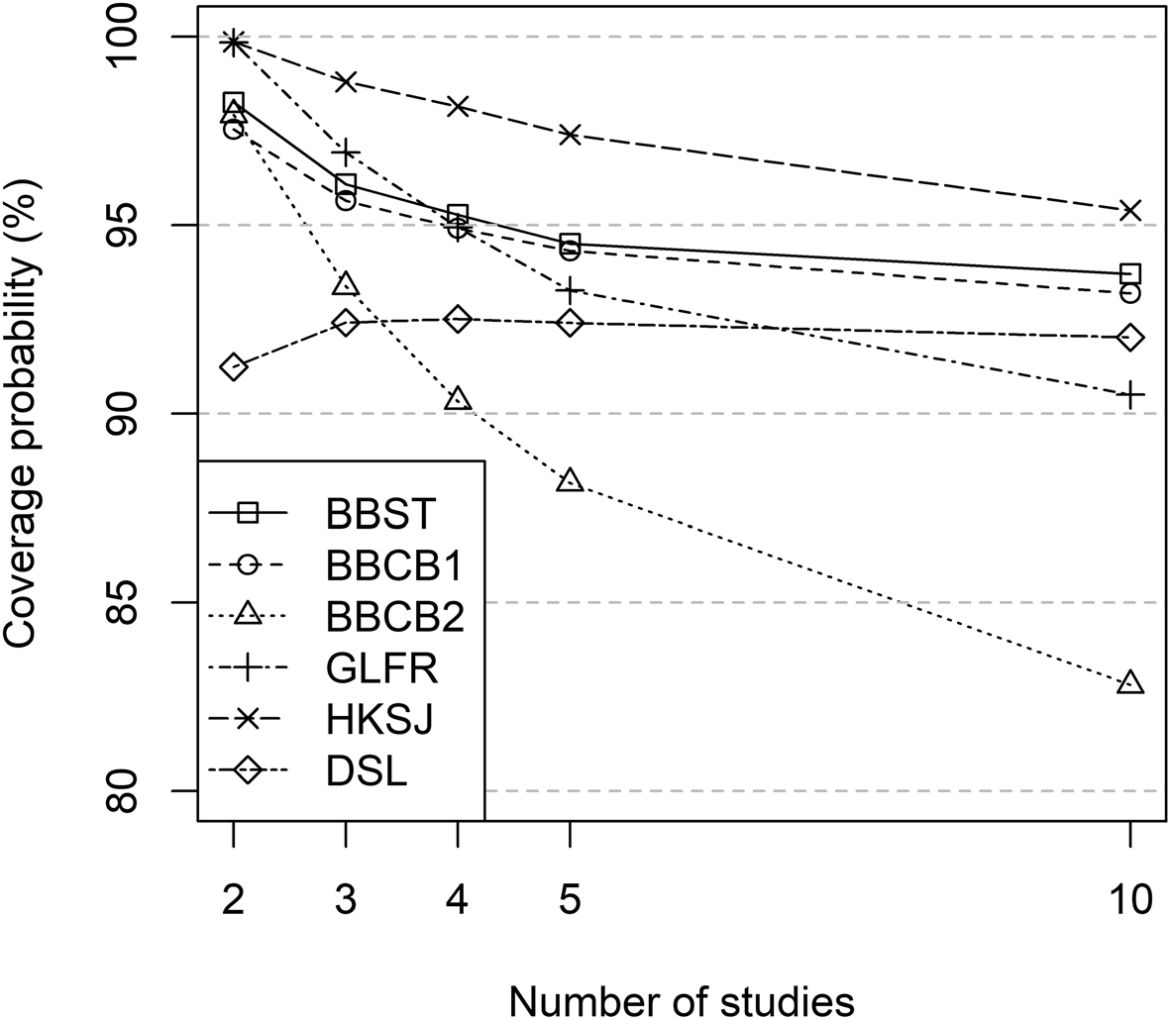
Coverage probability (%) for the OR under the alternative hypothesis (95% CI with 2K−2 degrees of freedom for BBST, BBCB1 and BBCB2)

Only HKSJ had coverage probabilities at or above 95% for all scenarios. BBCB2 had coverage probabilities < 95% for scenarios of 3 or more studies. The other BBMs (BBST, BBFR, BBCB1) had coverage probabilities at, above, or marginally below 95% for all scenarios. Coverage probabilities for GLFR were at or above 95% only for scenarios with 2, 3 or 4 studies. All other methods had coverage probabilities below 95% for all scenarios (GLRRI: ≤ 86.6%; DSL: ≤ 92.5%; MH: ≤ 82.2%; POR: ≤ 82.1%; COLL: ≤ 83.3%) (see Fig. 3 and Table S8 in the Supporting Information).

#### Length of 95% CI

Results for the length of the 95% CI were similar to results under the null hypothesis, with HKSJ and GLFR having the broadest intervals in all scenarios (see Table S9 in the Supporting Information).

#### Power

**Fig. 4:**
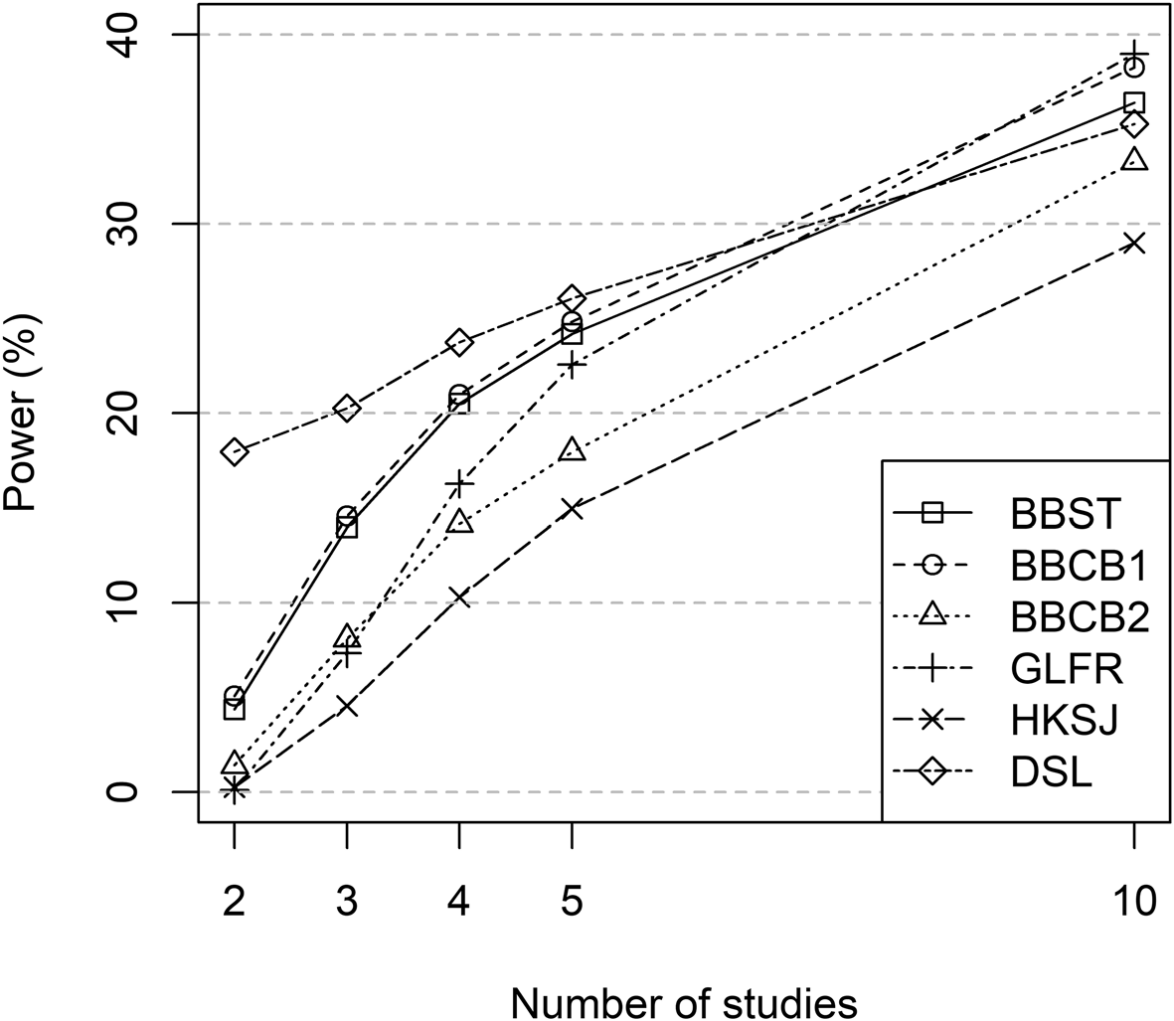
Power (%) for the OR under the alternative hypothesis (95% CI with 2K−2 degrees of freedom for BBST, BBCB1 and BBCB2)

Power for methods with coverage probability of ≥95% under the null hypothesis (BBST, BBFR, BBCB1, BBCB2, and HKSJ in all scenarios and GLFR in scenarios with 2, 3 and 4 studies) was quite low (still < 40% in scenarios with 10 studies). Power for BBMs (BBST, BBFR, BBCB1, BBCB2) was higher than for HKSJ in all scenarios. In scenarios with 2 studies, none of these methods showed a power > 5%, that is, no method yielded satisfactory results. In scenarios with 3 and 4 studies, power was still low (maximum 21.0% for BBCB1) but the differences in the methods became visible. Power was highest for BBST, BBFR and BBCB1, followed by BBCB2 and HKSJ with the lowest power (see Fig. 4 and Table S10 in the Supporting Information).

Methods with coverage probabilities < 95% under the null hypothesis (GLRRI, DSL, MH, POR, COLL) had higher power up to 55% (see Table S10 in the Supporting Information).

The small power values were to be expected due to the fact that the true ORs were near 1 (> 0.83) for about 25% of the simulations, the moderate sample sizes (mean: 185.7; median: 102.0), and only few studies in the MAs.

### Direct comparison of results for beta-binomial models

BBST and BBFR showed almost identical results. We assumed that one reason could be the maximum likelihood approximation method. Therefore, we tried to use another approximation method, the Gauss-Hermite quadrature with two quadratic points (QPOINTS = 2). However, in the case of a higher number of quadrature points, a floating point exception error occurred at one point, stopping the whole simulation. Therefore, we could not run the complete simulation using more quadrature points. We tried to reanalyse the simulated data using the Gauss-Hermite quadrature with 2 quadrature points for the BBFR. From the few existing results, it appears that BBST and BBFR vary the most if the ORs in the studies are very heterogeneous. Of notice, these were mainly data situations with strong heterogeneity (*P* value of Q test < 0.05).

The coverage probability of the BBCB2 was below the nominal level under the alternative hypothesis in scenarios with 3 or more studies. In contrast, BBCB1 performed well and very similarly to BBST considering all performance measures.

### Results for the relative risk

For the RR, we only considered BBST, GLFR, HKSJ, DSL and COLL in our simulation study. Because the results of BBST and BBFR were almost identical, we focussed on BBST. As mentioned earlier, it was not possible to compute RRs for BBCB1 and BBCB2. GLRRI and MH performed quite poorly considering the OR. Thus, we saw no need to reconsider them again. DSL and COLL also performed quite poorly, but we considered them due to their simplicity and as “benchmark” methods.

Results for RR were comparable to OR, except for the fact that BBST and GLFR struggled with convergence problems. BBST converged only in about 82% of each scenario (about 98% for OR) and GLFR between 90 and 95% (> 99% for OR). Noticeably, coverage probability for GLFR was higher and nearer the 95% level than that for OR (see Tables S11 – S20 in the Supporting Information).

### Sensitivity analysis

**Fig. 5:**
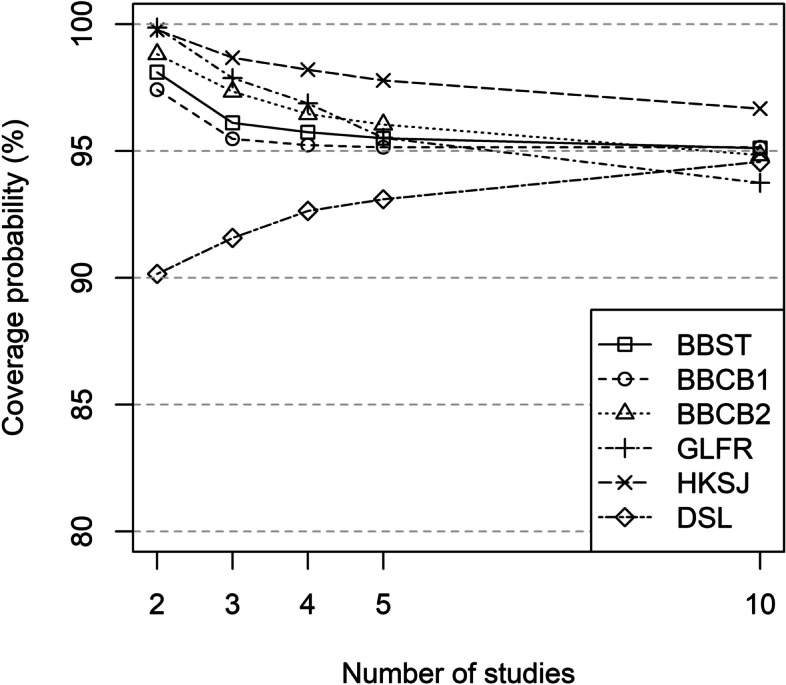
Coverage probability (%) for the OR under the null hypothesis in scenarios with no statistically significant heterogeneity (P value of Q test > 5%; 95% CI with 2K−2 degrees of freedom for BBST, BBCB1 and BBCB2)

The sensitivity analysis considering only data situations with no statistically significant heterogeneity (*P* value of Q test > 0.05) did not fundamentally alter the simulation results of the performance measures. The biggest influence was seen in coverage probabilities. Coverage probabilities below 95% increased and approached 95%. Coverage probabilities above 95% remained more or less stable. The biggest improvement was seen in MH, POR and COLL, but their coverage probabilities were still far below 95% (e.g. OR under H_0_ in scenarios with 4 studies: MH: 88.9%, POR: 88.4%, COLL: 88.9%). The most relevant improvement was shown in the GLFR, where coverage probabilities were far more near 95% in scenarios of 5 and 10 studies than in data situations where the appropriateness of pooling was not considered (see Fig. 5 as an example).

### Summary of results

The beta-binomial models BBST and BBCB1 (BBCB1 only for OR) performed well in scenarios with 3, 4, 5 and 10 studies. HKSJ was the only standard MA method that had adequate performance measures in these scenarios, although the coverage probability was very high (> 98%) for scenarios < 5 studies. In scenarios with only 2 studies, no method showed coverage probabilities near 95%; especially HKSJ and GLFR were far too conservative. Power was very low in this scenario; therefore, for 2 studies no method appeared appropriate. In scenarios with 3 and 4 studies, BBST, BBCB1 (BBCB1 only for OR), GLFR and HKSJ performed best, with the first two methods having higher power than the last two. In scenarios with 5 and 10 studies, BBST, BBCB1 (BBCB1 only for OR) and HKSJ performed best, with the first two methods having higher power than the last one.

## Discussion

We conducted a simulation study to compare BBMs with various standard meta-analytic methods in the case of very few studies. The BBST and the BBCB1 (BBCB1 only for OR) showed good results in the given data situations that were based on realistic data situations of Cochrane Reviews. The only standard MA method that showed acceptable results was HKSJ [39].

The attempt to extend the BBST by a random treatment effect term for the study, due to concerns about disrespecting the randomisation, failed. From the few results we obtained from the BBFR Gauss-Hermite quadrature, it could be seen that the 2 models varied the most in situations where a lot of heterogeneity between the study effects was present. As BBFR uses a random effect attached to the treatment effect in addition to the random intercept, this behaviour is to be expected, because only then “enough” additional heterogeneity remains to be estimated, i.e. not all heterogeneity goes into the random intercept. In practice, pooling will often not be appropriate in situations where even in the case of few studies there is such strong heterogeneity in the data. Thus, there is probably little benefit in using other approximation methods than Laplace in the case of sparse data, because either it has no impact on results, or it has only an impact in situations where there is a lot of heterogeneity. Our finding is in agreement with a recent study that assessed different approximations methods to perform meta-analyses using GLMMs in the case of rare events. This study found that the Gauss-Hermite quadrature is not superior to the Laplace approximation [38]. Thus, there seems to be no benefit in using BBFR in practical applications for meta-analyses of few studies. However, further research is necessary to obtain findings that are more conclusive.

Disrespecting the randomisation to a study arm of a specific study had no strong influence on the results of BBST and BBCB1. This was already pointed out more than 10 years ago [8] and is in line with a recent publication on arm-based (disrespecting randomisation) and contrast-based models (respecting randomisation) in network MA, where the authors conclude that both models are suitable for network MAs [40]. One reason for this is presumably that, especially in the case of few studies, heterogeneity cannot be estimated properly. As our simulation mirrors real MA situations, the problem may not be important in practice, as probably only a few data situations occur where this critical aspect of BBST and BBCB1 actually has negative consequences.

A very important point to keep in mind is the fact that we used adequate data situations based on RCT data. Our conclusions could be flawed when there is doubt about randomisation. Therefore, using another method respecting the randomisation to a study arm of a specific study as sensitivity analysis seems to be a reasonable approach. One could try a BBFR with more quadrature points than 1, but this might not work because of convergence problems. Thus, we recommend the use of standard procedures such as HKSJ in these situations. If the results and especially the point estimates are quite different, we would refrain from using BBST as a final method, because there probably is a problem with disrespecting the randomisation to a study arm of a specific study.

Surprisingly, BBCB2, which strictly respects randomisation, performed very badly regarding coverage probability under the alternative hypothesis. The narrow 95% CIs suggest that the standard errors are underestimated. This may be because only half of observations, concrete each study instead of each single group, contribute to the estimation of the parameters compared to BBCB1, where each arm of a study contributes to the estimation. Moreover, the assumption of homogeneity within one study may lead to overdispersion.

Although GLMMs are an intuitive alternative to the standard MA methods, GLFR and GLRRI performed quite poorly in many of our investigated scenarios. In Jackson et al. [5] both models (labelled as model 2 and 3) and their reparametrized versions were examined. In contrast to our results, the performance of GLFR was worse than that of GLRRI. Apart from this, the coverage probabilities for both models were below 95% for almost all 15 investigated scenarios, including 2 scenarios with 3 and 5 studies. The authors used maximum likelihood estimation based on adaptive quadrature with 7 (GLFR) and 1 quadrature point (GLRRI), whereas we used 1 quadrature point for each model. We do not think that the different number of quadrature points for GLFR can fully explain the differences in results. The noticeable difference in the simulation studies might be the difference in heterogeneity. Jackson et al. [5] simulated data with a median true *τ* for log OR of 0.024, whereas our median *τ* was $\sqrt{0.273}=0.52$, that is, considerably larger. As seen in setting 15 in Jackson et al. [5], both models performed worse if a lot of heterogeneity was present (coverage probabilities below 90%). Therefore, in scenarios with higher heterogeneity, the GLFR with 1 quadrature point can be more robust than with 7 points, leading to better performance measures. The opposite is true for the GLRRI. The greater the heterogeneity, the lesser the assumption of one random effect for the intercept may be justified, resulting in worse performance measures. In agreement with this assumption, the GLRRI often did not converge in the case of large heterogeneity, i.e. large values of *τ*^2^. As heterogeneity in the data is better detectable with an increasing number of studies in the MAs, this could also explain why, counterintuitively, the convergence of GLRRI decreases with an increasing number of studies. The partly differing results of these models in different simulation studies indicate how important the parameter settings in simulation studies could be, and thus stress the necessity of (independent) replications of results from simulation studies [41].

Some problems exist when using the RR in MAs [42, 43]. The problems arise from using the log link for the RR while the event probability *π* is bound to [0, 1] and are more pronounced if the event probabilities are large. Apart from problems with convergence, BBST and GLFR showed quite satisfactory results. A post-hoc analysis revealed that the main reason for the worse convergence were large baseline probabilities, namely values of *π*_*kC*_ > 0.5. In addition, in the case of *π*_*kC*_ < 0.5, high heterogeneity between the studies and small RRs led to non-converged simulations. Therefore, the log link affects the usability of these methods and further research is needed to improve the performance of these models.

An exact method for combining the effect sizes of the studies has recently been proposed [44]. This method was originally proposed for continuous data but could be easily implemented for binary data when using the logit or log link. Unfortunately, this method does not solve the problem of studies with no events in one or both study arms, because the effect size estimates of the studies are used to construct the 95% CI of the overall effect. Therefore, the same drawbacks exist with a continuous correction as with standard MA methods (HKSJ, DSL, MH for OR and RR).

Günhan et al. [45] investigated a Bayesian BBST. In a simulation study this model showed inappropriate coverage probabilities for very low OR (log OR < − 2), suggesting that BBMs are not suitable in situations where very large effects are expected. Because we tried to investigate realistic scenarios, such extreme values occurred in only a few simulations (≤ 10 in 10,000 simulations), which might be an explanation why no problems regarding coverage probability of the BBST were observed in our simulation study.

Our study has some limitations. As for all simulation studies, our results are only valid for data constellations we investigated. Because we based our simulation data on MAs actually performed in Cochrane Reviews, i.e. rather realistic scenarios, this is probably not a big problem. Another limitation is that we only investigated studies with balanced (1:1) randomisation schemes in the MA. In the case of a mixture of different randomisation schemes (1:1, 2:1, 3:1) the result of the BBST could be affected by the fact that the model disrespects randomisation. This highlights the importance of a sensitivity analysis using a method that respects randomisation.

Concerns could exist about the interpretability of the results with varying effect sizes under the alternative hypothesis. Therefore, we re-analysed the data by classifying the true effect sizes into four groups (< 1st quartile, between 1st quartile and median, between median and 3rd quartile, > 3rd quartile; data not shown). As expected, this had no impact on coverage probability. Likewise, for all other performance measures the values were bigger (smaller) for high effect size categories and smaller (bigger) for lower effect size categories. Thus, the given results can be interpreted as the mean/median of the different simulated effect sizes.

By chance, there were no double-zero studies in our MAs, but only single-zero studies. The number of MAs with at least one single-zero study varied from about 36% (2 studies) to 58% (10 studies). In an additional analysis (data not shown), the exclusion of MAs with single-zero studies led to similar results. However, this could be different if the number of single- and double-zero studies increases, which would require further investigation.

## Conclusion

In the case of very few (2 – 4) studies, the beta-binomial models BBST and BBCB1 (BBCB1 only for odds ratio) are valuable alternatives to standard random effects meta-analytic models, if the corresponding 95% confidence intervals for BBST and BBCB1 are constructed using the t distribution with 2*K* − 2 degrees of freedom.

For meta-analyses with 2 studies, no general recommendation for a specific model can be given due to very conservative coverage probabilities and very low power of all investigated methods. The application of a fixed effect model, if appropriate, or a qualitative summary of the study results could be a solution. For meta-analyses with 3 and 4 studies, the BBST and BBCB1 can be recommended in conjunction with a sensitivity analysis using HKSJ or another adequate method for a random effects model. For meta-analyses with 5 or more studies, the use of HKSJ is recommended. BBST and BBCB1 are useful methods for sensitivity analyses in this case.

## Supplementary Information

**Additional file 1: **Data simulation and parameter estimation.

**Additional file 2: Table S1.** Number of converged simulation runs (out of 10,000) for the odds ratio under the null hypothesis. **Table S2.** (Absolute) bias for the log odds ratio under the null hypothesis. **Table S3.** Coverage probability (%) for the odds ratio under the null hypothesis. **Table S4.** Length of 95% confidence interval for the log odds ratio under the null hypothesis. **Table S5.** Number of converged simulation runs (out of 10,000) for the odds ratio under the alternative hypothesis. **Table S6.** (Absolute) bias for the log odds ratio under the alternative hypothesis. **Table S7.** Percentage bias (%) for the log odds ratio under the alternative hypothesis. **Table S8.** Coverage probability (%) for the odds ratio under the alternative hypothesis. **Table S9.** Length of 95% confidence interval for the log odds ratio under the alternative hypothesis. **Table S10.** Power (%) for the odds ratio under the alternative hypothesis. **Table S11.** Number of converged simulation runs (out of 10,000) for the relative risk under the null hypothesis. **Table S12.** (Absolute) bias for the log relative risk under the null hypothesis. **Table S13.** Coverage probability (%) for the relative risk under the null hypothesis. **Table S14.** Length of 95% confidence interval for the log relative risk under the null hypothesis. **Table S15.** Number of converged simulation runs (out of 10,000) for the relative risk under the alternative hypothesis. **Table S16.** (Absolute) bias of the log relative risk under the alternative hypothesis. **Table S17.** Percentage bias (%) for the log relative risk under the alternative hypothesis. **Table S18.** Coverage probability (%) for the relative risk under the alternative hypothesis. **Table S19.** Length of 95% confidence interval for the log relative risk under the alternative hypothesis. **Table S20.** Power (%) for the relative risk under the alternative hypothesis.

## Data Availability

All data generated in this study are available as supplementary material.

## Abbreviations

BBM: Beta-binomial model
BBST: Standard (“common-rho”) beta-binomial model
BBFR: Standard beta-binomial model with an additional random treatment effect
BBCB: “common-beta” beta-binomial model
COLL: Collapsed table
DSL: DerSimonian-Laird
GLMM: Generalised linear mixed model
GLFR: Generalised linear mixed model with a fixed intercept and random treatment effect
GLRR: Generalised linear mixed model with a random intercept and random treatment effect
HKSJ: Hartung-Knapp-Sidik-Jonkman
IV-REM: Inverse variance random effects model
MA: Meta-analysis
MH: Mantel-Haenszel
OR: Odds ratio
POR: Peto odds ratio
RCT: Randomised controlled trial
RR: Relative risk
