# Supplementary material for: Performance of several types of beta-binomial models in comparison to standard approaches for meta-analyses with very few studies
Source: BMC Med Res Methodol. 2022 Dec 13;22:319. doi: 10.1186/s12874-022-01779-3 (PMC9745934; doi:10.1186/s12874-022-01779-3)
Supplement: Supplementary file 1 — Additional file 1: Data simulation and parameter estimation. [file 12874_2022_1779_MOESM1_ESM.docx]

| **Data simulation and parameter estimation** |
| --- |

* SIMULATION_FEW STUDIES_one stage.SAS (22.11.2019)

This program performs a simulation study for assessing the performance of beta-binomial regression models with random effects and GLM models in comparison to the

standard beta-binomial and two-stage meta-analyis models for very low number of studies.

It introduces the SAS macro %FewStudiesOneStageSim2 which performs the simulation (data generation, computation of starting values, parameter estimation, and summarization

of results in a single data set) for the simulation scenarios. A single simulation scenario is defined by the macro parameters

effect : =1 H1 Generate ORs

: =0 H0 No effect OR=1

rem : =1 Generate data from the standard random effects model

=0 Generate data from the standard fixed effects model

nsimruns : Number of generated meta-analyses

nstudy : Number of studies in a single meta-analysis

seedsim : Initializing value of the random number generator

printsimdata : =1 Print the simulated data

=0 (Default) Do not print the simulated data

options pagesize=60 linesize=120;

libname result "C:\Users\tmathes\Desktop\SAS";

**%macro** FewStudiesSim2(nstudy=,effect=,rem=,seedsim=,nsimruns=,printsimdata=**0**);

******************************************************************************************;

*** PRELIMINARIES **************************************************************************;

******************************************************************************************;

options nomlogic nomprint nosource nonotes nosymbolgen;

proc printto log="C:\Users\tmathes\Desktop\SAS\Log_&seedsim..txt"

print="C:\Users\tmathes\Desktop\SAS\Output_&seedsim..txt";

run;

proc format;

value treatf **1**=" Treatment" **0**="Control";

value eventf **1**=" Yes" **0**="No";

value $ident_t 'H0'='No treatment effect' 'H1'='Average treatment effect';

run;

*****************************************************************************************************************************************************************************;

*****************************************************************************************************************************************************************************;

********** DATA GENERATION **************************************************************************************************************************************************;

*****************************************************************************************************************************************************************************;

*****************************************************************************************************************************************************************************;

* The data set SIMDATA contains the simulated data sets, the variable SIMRUNS indexes the single simulation run;

data simdata;

* Initialize random seed;

call streaminit(&seedsim);

************************** Loop across the number of simulation runs ***************************************************************;

do simruns=**1** to &nsimruns;

***** Begin data generation ******************************************************************************************************;

* ... Declare number of studies from the macro variable &nstudy;

NumberofStudies=&nstudy;

* Generate a baseline event probability from a beta distribution with alpha=0.393628 und beta=0.881246.

These values originate from an analysis of the 14,886 meta-analyses with binary outcomes from Rebecca Turner

(vgl. Analyse_Baseline_Risks_Turner.SAS)

A decent fit results from fitting a beta distribution with alpha=0.422932 and beta=1.433449. To be concrete,

comparing observed (from Turner) vs. fitted summary statistics, we find for the

Median 0.1262 vs. 0.1325, for the

Mean 0.2271 vs. 0.2336 and for the

standard deviation 0.2558 vs. 0.2556

The true mean and standard deviation from a Beta(0.422932, 1.433449) distribution are 0.22783 and 0.24817

ATTENTION: The fact that the baseline probabilities are simulated from a beta distribution DOES NOT MEAN that the

data are generated from the beta-binomial model. We here generate one fixed baseline probability for a single meta-analysis,

whereas the beta-binomial model assumes that the baseline probabilities OF THE STUDIES WITHIN THE META-ANALYSIS follow

a beta distribution.

The true model is still a standard inverse-variance random-effects model;

true_pc=rand('BETA',**0.422932**,**1.433449**);

if true_pc<**0.01** then do; true_pc=**0.01**; end;

true_logit_pc=log(true_pc/(**1**-true_pc));

* For the H0 situation, the true OR is set to 1;

%if &effect=**0** %then %do;

true_OR=**1**;

true_LogOR=**0**;

%end;

* For the H1 situation, the ORs have to be generated;

%if &effect=**1** %then %do;

* Generation of the effects (ORs);

LogNormal_mean_Turner_OR= -**0.59**;LogNormal_stddev_Turner_OR=**0.61**;

* Generation of a skewed distribution using Fleishmans power transformation method;

random_standard_normal_OR=rand("NORMAL");

random_skewed_normal_OR = **0.659546319** + **0.659546319***random_standard_normal_OR

-**0.228926754***random_standard_normal_OR****2** - **0.087359908***random_standard_normal_OR****3**;

true_LogOR=LogNormal_mean_Turner_OR + LogNormal_stddev_Turner_OR*random_skewed_normal_OR;

if true_OR<**0.95** then do;

true_OR=exp(true_LogOR);end;

if true_OR>**0.95** then do;

LogNormal_mean_Turner_OR= -**0.59**;LogNormal_stddev_Turner_OR=**0.61**;

* Generation of a skewed distribution using Fleishmans power transformation method;

random_standard_normal_OR=rand("NORMAL");

random_skewed_normal_OR = **0.659546319** + **0.659546319***random_standard_normal_OR

-**0.228926754***random_standard_normal_OR****2** - **0.087359908***random_standard_normal_OR****3**;

true_LogOR=LogNormal_mean_Turner_OR + LogNormal_stddev_Turner_OR*random_skewed_normal_OR;

true_OR=exp(true_LogOR); end;

if true_OR>**0.95** then do;

LogNormal_mean_Turner_OR= -**0.59**;LogNormal_stddev_Turner_OR=**0.61**;

* Generation of a skewed distribution using Fleishmans power transformation method;

random_standard_normal_OR=rand("NORMAL");

random_skewed_normal_OR = **0.659546319** + **0.659546319***random_standard_normal_OR

-**0.228926754***random_standard_normal_OR****2** - **0.087359908***random_standard_normal_OR****3**;

true_LogOR=LogNormal_mean_Turner_OR + LogNormal_stddev_Turner_OR*random_skewed_normal_OR;

true_OR=exp(true_LogOR); end;

if true_OR>**0.95** then do;

LogNormal_mean_Turner_OR= -**0.59**;LogNormal_stddev_Turner_OR=**0.61**;

* Generation of a skewed distribution using Fleishmans power transformation method;

random_standard_normal_OR=rand("NORMAL");

random_skewed_normal_OR = **0.659546319** + **0.659546319***random_standard_normal_OR

-**0.228926754***random_standard_normal_OR****2** - **0.087359908***random_standard_normal_OR****3**;

true_LogOR=LogNormal_mean_Turner_OR + LogNormal_stddev_Turner_OR*random_skewed_normal_OR;

true_OR=exp(true_LogOR); end;

if true_OR>**0.95** then do;

LogNormal_mean_Turner_OR= -**0.59**;LogNormal_stddev_Turner_OR=**0.61**;

* Generation of a skewed distribution using Fleishmans power transformation method;

random_standard_normal_OR=rand("NORMAL");

random_skewed_normal_OR = **0.659546319** + **0.659546319***random_standard_normal_OR

-**0.228926754***random_standard_normal_OR****2** - **0.087359908***random_standard_normal_OR****3**;

true_LogOR=LogNormal_mean_Turner_OR + LogNormal_stddev_Turner_OR*random_skewed_normal_OR;

true_OR=exp(true_LogOR); end;

if true_OR>**0.95** then do;

true_LogOR=log(**0.684**); true_OR=**0.684**;

end;

%end;

* For the random effects situation, the tau-squares have to be generated;

%if &rem=**1** %then %do;

* Generation of the random effects variance (tau-square);

LogNormal_mean_Turner_Tau=-**1.47**;LogNormal_stddev_Turner_Tau=**1.65**;

* Generation of a skewed distribution using Fleishmans power transformation method;

random_standard_normal_tau=rand("NORMAL");

random_skewed_normal_tau = **0.104796973** + **1.049992872***random_standard_normal_tau

-**0.104796973***random_standard_normal_tau****2** - **0.020782441***random_standard_normal_tau****3**;

Log_TauSquare_skewed=LogNormal_mean_Turner_Tau + LogNormal_stddev_Turner_Tau*random_skewed_normal_tau;

true_TauSquare=exp(Log_TauSquare_skewed);

%end;

******************** Loop across the number of studies within a meta-analysis ***************************************************;

do study=**1** to NumberofStudies;

* Generate the sample size for the two groups following the distribution in Turner et al., Table 2;

LogNormal_mean_Turner_SSize=**4.615**;LogNormal_stddev_Turner_SSize=**1.1**;

Log_StudySize=LogNormal_mean_Turner_SSize + LogNormal_stddev_Turner_SSize*rand('NORMAL');

* Determine the study size by doubling the "ceiled" group size, where ceiling means

calculating the smallest integer that is greater than or equal to the argument;

StudySize=**1**+ceil(exp(Log_StudySize));

if StudySize<**20** then do; Studysize=**20**; end;

* In the random effects situation, the true event probabilities are varying according to the random Log Odds Ratio;

%if &rem=**1** %then %do;

true_TauSquare_i=true_TauSquare*rand('NORMAL');

* Calculate the study-specific event probability in the treatment group on the expit scale.

The linear predictor here is the sum of the logit of the event probability in the control group, the true LogOR und the random tau-square in the

respective study;

true_pt_i=exp(true_logit_pc + true_LogOR + true_TauSquare_i)/(**1**+exp(true_logit_pc + true_LogOR + true_TauSquare_i));

%end;

%if &rem=**0** %then %do;

true_pt=exp(true_logit_pc + true_LogOR)/(**1**+exp(true_logit_pc + true_LogOR));

%end;

* Simulate the sample size in the treatment group by mimicking a randomization process with

a binomial probability 0.5;

nt=rand('BINOMIAL',**0.5**,StudySize);

nc=StudySize-nt;

* Exclude extrem cases with <10 treated observations in a treatment group;

if nt<**10** then do; nt=**10**; end;

if nc<**10** then do; nc=**10**; end;

* Generate the number of events in both groups;

sc=**0**; st=**0**;

do while(sc+st=**0**);

put sc=; put st=;

sc=rand('BINOMIAL',true_pc,nc);

%if &rem=**0** %then %do;st=rand('BINOMIAL',true_pt,nt); %end;

%if &rem=**1** %then %do;st=rand('BINOMIAL',true_pt_i,nt); %end;

end;

***** End of data generation *****************************************************************************************************;

* Estimate raw event probabilities;

pt=st/nt; pc=sc/nc;

nsucc=st+sc;

ntrials=nt+nc;

p_overall=nsucc/ntrials;

test_OR=(pt*(**1**-pc))/(pc*(**1**-pt));

test_LogOR=log(test_OR);

weight_test_LogOR=**1**/(**1**/st + **1**/(nt-st) + **1**/sc + **1**/(nc-sc));

* Generate a pseudo intercept for each study;

int=**1**;

output;

end; * of loop across studies within a meta-analysis;

end; * of loop across the number of simulation runs;

run;

%if &printsimdata=**1** %then %do;

proc print data=simdata;

title"simdata";

run;

%end;

* Control data generation;

* ods select none;

/*proc means data=simdata n mean std median min q1 q3 max;

%if &rem=1 %then %do; var nt nc StudySize st sc pt pc test_OR true_pc true_OR true_LogOR true_TauSquare_i true_pt_i; %end;

%if &rem=0 %then %do; var nt nc StudySize st sc pt pc test_OR true_pc true_OR true_LogOR; %end;

title"Control generation of random effects data";

run;*/

* ods select all;

* Define a id (IDENT) for the simulation scenarios;

data ident;

%if &effect = **0** %then %do;ident_t='H0';%end;

%if &effect = **1** %then %do;ident_t='H1';%end;

%if &rem = **0** %then %do;ident_REM='FEM';%end;

%if &rem = **1** %then %do;ident_REM='REM';%end;

%if &nstudy=**2** %then %do; ident_nstudy='02';%end;

%if &nstudy=**3** %then %do; ident_nstudy='03';%end;

%if &nstudy=**4** %then %do; ident_nstudy='04';%end;

%if &nstudy=**5** %then %do; ident_nstudy='05';%end;

%if &nstudy=**10** %then %do; ident_nstudy='10';%end;

Ident = ident_t || ident_REM || ident_nstudy;

call symput("Ident",Ident);

run;

%put Ident: &Ident;

* Reorganize SIMDATA, so that each single study has two observations (one for treatment, one for control, SIMDATADOUBLE) or

each individual constitutes a single observation (SIMDATAEXPLODED);

data simdatadescriptive;

set simdata(keep=simruns true_LogOR);

by simruns;

if first.simruns;

run;

/*proc print data=simdatadescriptive;title"simdatadescritptive"; run;*/

data simdatadouble_temp1;

set simdata(keep=simruns study st sc nt nc);

by simruns;

do k=**1** to **2**;output;end;

run;

data simdatadouble(keep=simruns study treatment control success n dummy);

set simdatadouble_temp1;

by simruns;

if mod(k,**2**)=**1** then do; treatment=**1**; control=**0**; success=st; n=nt; end;

if mod(k,**2**)=**0** then do; treatment=**0**; control=**1**; success=sc; n=nc; end;

dummy=**1**;

run;

/*proc print data=simdatadouble;title"simdatadouble";run;*/

* Data for the Guimaraes model;

data simdatahelp;

set simdata(keep=simruns study st sc nt nc);

by simruns;

do i=**1** to **4**; output; end;

dummy=**1**;

run;

data simdataquadruple;

set simdatahelp;

by simruns;

if i=**1** then do; success=st; treatment=**1**;indicator=**1**;CountregTreatment=treatment*(indicator=**1**);studygroup=study***2**-**1**;end;

if i=**2** then do; success=nt-st; treatment=**1**;indicator=**0**;CountregTreatment=treatment*(indicator=**1**);studygroup=study***2**-**1**;end;

if i=**3** then do; success=sc; treatment=**0**;indicator=**1**;CountregTreatment=treatment*(indicator=**1**);studygroup=study***2**;end;

if i=**4** then do; success=nc-sc; treatment=**0**;indicator=**0**;CountregTreatment=treatment*(indicator=**1**);studygroup=study***2**;end;

run;

/*proc print data=simdataquadruple;title"simdataquadruple";run;*/

*Data for the inverse-variance models (with continuity correction);

data simdatacontinuity(keep=simruns study st nt sc nc true_OR test_OR test_LogOR weight_test_LogOR int);

set simdata;

by simruns;

if st=**0** then do; st=**0.5**; sc=sc+**0.5**; end;

if sc=**0** then do; sc=**0.5**; st=st+**0.5**; end;

pt=st/nt; pc=sc/nc;

test_OR=(pt*(**1**-pc))/(pc*(**1**-pt));

test_LogOR=log(test_OR);

weight_test_LogOR=**1**/(**1**/st + **1**/(nt-st) + **1**/sc + **1**/(nc-sc));

run;

/*proc print data=simdatacontinuity;title"simdatacontinuity";run;*/

*Data for Mantel-Haenszel analysis and collapsed table;

data simdataexplode(drop=l);

set simdatadouble;

by simruns;

do l=**1** to n;

if l <= success then event=**1**;

if l > success then event=**0**;

status=**2**-event;

output;

end;

run;

/*proc print data=simdataexplode;title"simdataexplode";run;*/

******************************************************************************************;

*** STARTING VALUES ********************************************************************;

******************************************************************************************;

* Compute means, standard deviations for the estimated event probabilities in both treatment groups;

ods select none;

proc corr data=simdata pearson cov;

var pt;with pc;

by simruns;

ods output SimpleStats=SimpleStats_pt(keep=Simruns Variable Mean StdDev where=(Variable="pt") rename=(mean=start_pt StdDev=start_stddev_pt))

SimpleStats=SimpleStats_pc(keep=Simruns Variable Mean StdDev where=(Variable="pc") rename=(mean=start_pc StdDev=start_stddev_pc));

run;

ods select all;

/*proc print data=SimpleStats_pc;run;*/

ods select none;

proc means data=simdata mean stddev;

var test_LogOR;

by simruns;

output out=SimpleStats_OR mean=start_LogOR StdDev=start_stddev_LogOR;

run;

ods select all;

/*proc print data=SimpleStats_OR;run;*/

* Starting values for the Beta-Binomial-Models;

data startingvalues_Beta_Binomial(keep=simruns rho b0 b_treat sigma);

merge SimpleStats_pt(keep=simruns start_pt start_stddev_pt)

SimpleStats_pc(keep=simruns start_pc start_stddev_pc)

SimpleStats_OR(keep=simruns start_LogOR start_stddev_LogOR);

* Pooling variances and raw probabilities;

if start_stddev_pt=**0** then do; start_stddev_pt=**0.001**;end;

if start_stddev_pc=**0** then do; start_stddev_pc=**0.001**;end;

if start_pt=**0** then do; start_pt=**0.001**;end;

if start_pc=**0** then do; start_pc=**0.001**;end;

pooledvar=(start_stddev_pt****2** + start_stddev_pc****2**)/**2**;

pooledp =(start_pt+start_pc)/**2**;

rho=pooledvar/(pooledp*(**1**-pooledp))/(**1**-pooledvar/(pooledp*(**1**-pooledp)));

logitrho=log(rho/(**1**-rho));

b0 =log(start_pc/(**1**-start_pc));

if (start_pt=**0**) or (start_pc=**0**) then do; b_treat=**0**; end;

else do; b_treat=log((start_pt*(**1**-start_pt))/(start_pc*(**1**-start_pc))); end;

mu=exp((b0+b_treat)/(**1**+exp(b0+b_treat)));

alpha=mu*((**1**-exp(logitrho))/(exp(logitrho)));

beta=(**1**-mu)*(**1**-exp(logitrho))/exp(logitrho);

sigma=start_stddev_LogOR;

by simruns;

run;

/*proc print data=startingvalues_Beta_Binomial;title"startingvalues_Beta_Binomial";run;*/

*****************************************************************************************************************************************************************************;

*****************************************************************************************************************************************************************************;

********** ANALYSIS ********************************************************************************************************************************************************;

*****************************************************************************************************************************************************************************;

*****************************************************************************************************************************************************************************;

* Naming conventions:

Meta-analysis Models

BBST1 : Standard Beta-Binomialmodell with t-distributed confidence intervals using K-1 degrees of freedom

BBST2 : Standard Beta-Binomialmodell with t-distributed confidence intervals using Kx2-2 degrees of freedom

BBFR1 : Beta-binomial regression with fixed intercept and random treatment effect with K-1 degrees of freedom

BBFR2 : Beta-binomial regression with fixed intercept and random treatment effect with Kx2-2 degrees of freedom

BBGU1 : Beta-Binomialmodell according Guimaraes with t-distributed confidence intervals using K-1 degrees of freedom

BBGU2 : Beta-Binomialmodell according Guimaraes with t-distributed confidence intervals using Kx2-2 degrees of freedom

BBCB1 : Common-beta Beta-Binomialmodell (breaks the randomisation) with t-distributed confidence intervals using K-1 degrees of freedom

BBCB2 : Common-beta Beta-Binomialmodell (breaks the randomisation) with t-distributed confidence intervals using Kx2-2 degrees of freedom

GLFR_ : Generalised linear mixed model with fixed intercept and random treatment effect

GLRRI : Generalised linear mixed model with random intercept and random treatment effect (indepedent)

HKPM_ : Knapp-Hartung-Method with variance correction and Paule-Mandel-heterogenity variance estimator

MHKPM : Knapp-Hartung-Method without variance correction and Paule-Mandel-heterogenity variance estimator

DLRE_ : DerSimonian-Laird-Method

MAHA_ : Mantel-Haenszel-Method

PETO_ : Peto-Method

COLLA : Collapsed Table

Treatment effect measure:

OR: Odds ratio

*****************************************************************************************************************************;

*** EFFECT ESTIMATION *******************************************************************************************************;

*****************************************************************************************************************************;

**********************************************************************************************************;

*** Beta-binomial models *********************************************************************************;

**********************************************************************************************************;

***Standard Beta-binomial-Modell von Kuss;

ods select none;

proc nlmixed data=simdatadouble tech=NRRIDG;

by simruns;

parms / bydata data=startingvalues_Beta_Binomial;

bounds **0.00001**<=rho<=**0.99999**;

mu= exp(b0 + b_treat*treatment)/(**1** + exp(b0 + b_treat*treatment));

alpha=mu*(**1**-rho)/rho;

beta=(**1**-mu)*(**1**-rho)/rho;

ll= lgamma(n+**1**)+lgamma(success+alpha)+lgamma(n-success+beta)+lgamma(alpha+beta)

-lgamma(success+**1**)-lgamma(n-success+**1**)-lgamma(n+alpha+beta) -lgamma(alpha)-lgamma(beta);

model success ~ general(ll);

* Estimate the treatment effect;

estimate "LogOR" b_treat;

ods output AdditionalEstimates=LogOR_BBST_temp(where=(Label="LogOR") rename=(Estimate=LogOR_BBST StandardError=SE_LogOR_BBST));

title"Beta-binomial model, Kuss";

run;

ods select all;

* Confidence intervals with k-1 degrees of freedom;

data LogOR_BBST1(keep=simruns LogOR_BBST1 CI95L_LogOR_BBST1 CI95U_LogOR_BBST1);

set LogOR_BBST_temp;

LogOR_BBST1=LogOR_BBST;

CI95L_LogOR_BBST1 = LogOR_BBST1 - quantile('T',**.975**,&nstudy-**1**)* SE_LogOR_BBST;

CI95U_LogOR_BBST1 = LogOR_BBST1 + quantile('T',**.975**,&nstudy-**1**)* SE_LogOR_BBST;

run;

/*proc print data=LogOR_BBST1;run;*/

* Confidence intervals with k-1 degrees of freedom;

data LogOR_BBST2(keep=simruns LogOR_BBST2 CI95L_LogOR_BBST2 CI95U_LogOR_BBST2);

set LogOR_BBST_temp;

LogOR_BBST2=LogOR_BBST;

CI95L_LogOR_BBST2 = LogOR_BBST2 - quantile('T',**.975**,&nstudy***2**-**2**)* SE_LogOR_BBST;

CI95U_LogOR_BBST2 = LogOR_BBST2 + quantile('T',**.975**,&nstudy***2**-**2**)* SE_LogOR_BBST;

run;

/*proc print data=LogOR_BBST2;run;*/

* Beta-binomial-Modell mit zufälligem Behandlungseffekt;

ods select none;

proc nlmixed data=simdatadouble tech=NRRIDG noad qpoints=**1**;

by simruns;

parms / bydata data=startingvalues_Beta_Binomial;

bounds **0.00001**<=rho<=**0.99999**;

mu=exp(b0 + (ut+b_treat)*treatment)/(**1** + exp(b0 + (ut+b_treat)*treatment));

alpha=mu*(**1**-rho)/rho;

beta=(**1**-mu)*(**1**-rho)/rho;

ll= lgamma(n+**1**)+lgamma(success+alpha)+lgamma(n-success+beta)+lgamma(alpha+beta)

-lgamma(success+**1**)-lgamma(n-success+**1**)-lgamma(n+alpha+beta) -lgamma(alpha)-lgamma(beta);

model success ~ general(ll);

random ut ~ normal(**0**, sigma****2**) subject=study;

estimate "LogOR" b_treat;

ods output AdditionalEstimates=LogOR_BBFR_temp(where=(Label="LogOR") rename=(Estimate=LogOR_BBFR StandardError=SE_LogOR_BBFR));

title"Beta-binomial model, fixed study effect and random treatment effect";

run;

ods select all;

* Confidence intervals with k-1 degrees of freedom;

data LogOR_BBFR1(keep=simruns LogOR_BBFR1 CI95L_LogOR_BBFR1 CI95U_LogOR_BBFR1);

set LogOR_BBFR_temp;

LogOR_BBFR1=LogOR_BBFR;

CI95L_LogOR_BBFR1 = LogOR_BBFR1 - quantile('T',**.975**,&nstudy-**1**)* SE_LogOR_BBFR;

CI95U_LogOR_BBFR1 = LogOR_BBFR1 + quantile('T',**.975**,&nstudy-**1**)* SE_LogOR_BBFR;

run;

/*proc print data=LogOR_BBFR1;run;*/

* Confidence intervals with kx2-2 degrees of freedom;

data LogOR_BBFR2(keep=simruns LogOR_BBFR2 CI95L_LogOR_BBFR2 CI95U_LogOR_BBFR2);

set LogOR_BBFR_temp;

LogOR_BBFR2=LogOR_BBFR;

CI95L_LogOR_BBFR2 = LogOR_BBFR2 - quantile('T',**.975**,&nstudy***2**-**2**)* SE_LogOR_BBFR;

CI95U_LogOR_BBFR2 = LogOR_BBFR2 + quantile('T',**.975**,&nstudy***2**-**2**)* SE_LogOR_BBFR;

run;

/*proc print data=LogOR_BBFR2;run;*/

***Beta-binomial-Modell von Guimaraes*******************************************************************;

ods select none;

proc countreg data=simdataquadruple groupid=study;

by simruns;

model success=indicator CountregTreatment/ errorcomp=fixed dist=negbin;

ods output ParameterEstimates=LogOR_BBGU_temp(where=(Parameter="CountregTreatment") rename=(Estimate=LogOR_BBGU StdErr=SE_LogOR_BBGU));

title"Beta-binomial model, Guimaraes";

run;

ods select all;

* Confidence intervals with k-1 degrees of freedom;

data LogOR_BBGU1(keep=simruns LogOR_BBGU1 CI95L_LogOR_BBGU1 CI95U_LogOR_BBGU1);

set LogOR_BBGU_temp;

LogOR_BBGU1=LogOR_BBGU;

CI95L_LogOR_BBGU1 = LogOR_BBGU1 - quantile('T',**.975**,&nstudy-**1**)* SE_LogOR_BBGU;

CI95U_LogOR_BBGU1 = LogOR_BBGU1 + quantile('T',**.975**,&nstudy-**1**)* SE_LogOR_BBGU;

run;

/*proc print data=LogOR_BBGU1;run;*/

* Confidence intervals with kx2-2 degrees of freedom;

data LogOR_BBGU2(keep=simruns LogOR_BBGU2 CI95L_LogOR_BBGU2 CI95U_LogOR_BBGU2);

set LogOR_BBGU_temp;

LogOR_BBGU2=LogOR_BBGU;

CI95L_LogOR_BBGU2 = LogOR_BBGU2 - quantile('T',**.975**,&nstudy***2**-**2**)* SE_LogOR_BBGU;

CI95U_LogOR_BBGU2 = LogOR_BBGU2 + quantile('T',**.975**,&nstudy***2**-**2**)* SE_LogOR_BBGU;

run;

/*proc print data=LogOR_BBGU2;run;*/

***Common-beta Beta-binomial-Modell *******************************************************************;

ods select none;

proc countreg data=simdataquadruple groupid=studygroup;

by simruns;

model success=indicator CountregTreatment/ errorcomp=fixed dist=negbin;

ods output ParameterEstimates=LogOR_BBCB_temp(where=(Parameter="CountregTreatment") rename=(Estimate=LogOR_BBCB StdErr=SE_LogOR_BBCB));

title"Beta-binomial model, Common-beta";

run;

ods select all;

* Confidence intervals with k-1 degrees of freedom;

data LogOR_BBCB1(keep=simruns LogOR_BBCB1 CI95L_LogOR_BBCB1 CI95U_LogOR_BBCB1);

set LogOR_BBCB_temp;

LogOR_BBCB1=LogOR_BBCB;

CI95L_LogOR_BBCB1 = LogOR_BBCB1 - quantile('T',**.975**,&nstudy-**1**)* SE_LogOR_BBCB;

CI95U_LogOR_BBCB1 = LogOR_BBCB1 + quantile('T',**.975**,&nstudy-**1**)* SE_LogOR_BBCB;

run;

/*proc print data=LogOR_BBCB1;run;*/

* Confidence intervals with kx2-2 degrees of freedom;

data LogOR_BBCB2(keep=simruns LogOR_BBCB2 CI95L_LogOR_BBCB2 CI95U_LogOR_BBCB2);

set LogOR_BBCB_temp;

LogOR_BBCB2=LogOR_BBCB;

CI95L_LogOR_BBCB2 = LogOR_BBCB2 - quantile('T',**.975**,&nstudy***2**-**2**)* SE_LogOR_BBCB;

CI95U_LogOR_BBCB2 = LogOR_BBCB2 + quantile('T',**.975**,&nstudy***2**-**2**)* SE_LogOR_BBCB;

run;

/*proc print data=LogOR_BBCB2;run;*/

**********************************************************************************************************;

*** GLMM models ******************************************************************************************;

**********************************************************************************************************;

***GLMM Modell mit zufälligen Behandlungseffekt (random treatment effect only)*************************;

ods select none;

proc glimmix data=simdatadouble order=formatted method=quad(qpoints=**1**);

by simruns;

class study treatment;

model success/n=study treatment / d=bin link=logit solution cl ddf=**10000**;

random treatment / subject=study;

nloptions tech=NRRIDG;

ods output ParameterEstimates=LogOR_GLFR_temp(where=(treatment=**1**)

rename=(Estimate=LogOR_GLFR_ StdErr=SE_LogOR_GLFR_

Lower=CI95L_LogOR_GLFR_ Upper=CI95U_LogOR_GLFR_));

format treatment treatf.;

title"GLMM, fixed study effect and random treatment effect";

run;

ods select all;

data LogOR_GLFR_(keep=simruns LogOR_GLFR_ CI95L_LogOR_GLFR_ CI95U_LogOR_GLFR_);

set LogOR_GLFR_temp;

run;

/*proc print data=LogOR_GLFR_;run;*/

***GLMM Modell mit unabhängigen zufälligem Effekt über die Studien und zufälligem Behandlungseffekt(random intercept and random treatment effect [indepedent]);

ods select none;

proc glimmix data=simdatadouble order=formatted method=quad(qpoints=**1**);

by simruns;

class study treatment;

model success/n=treatment / d=bin link=logit solution cl ddf=**10000**;

random intercept treatment / subject=study type=vc;

nloptions tech=NRRIDG;

ods output ParameterEstimates=LogOR_GLRRI_temp(where=(treatment=**1**)

rename=(Estimate=LogOR_GLRRI StdErr=SE_LogOR_GLRRI

Lower=CI95L_LogOR_GLRRI Upper=CI95U_LogOR_GLRRI));

format treatment treatf.;

title"GLMM, random study effect and random treatment effect (indepedent)";

run;

ods select all;

data LogOR_GLRRI(keep=simruns LogOR_GLRRI CI95L_LogOR_GLRRI CI95U_LogOR_GLRRI);

set LogOR_GLRRI_temp;

run;

/*proc print data=LogOR_GLRR_;run;*/

**********************************************************************************************************;

*** Standard models **************************************************************************************;

**********************************************************************************************************;

****Knapp-Hartung-Verfahren mit Paule Mandel Heterogenitätsschätzer*********************************************************************;

*prepare dataset for Paule and Mandel Macro;

data pmivre;

set simdatacontinuity;

vari=**1**/weight_test_LogOR;

run;

* Determine the number of studies;

ods select none;

proc means data=pmivre n;

var study;

by simruns;

output out=outmeans n(study)=nstudies;

run;

ods select all;

* ... and merge with the original data set;

data pmstart (keep=simruns nstudies study test_LogOR vari);

merge outmeans pmivre;

by simruns;

run;

*macro to estimate the Paule and Mandel heterogenity variance etimator Tau(pm);

**%macro** PmEstimator(data=, start_tau_square_pm=, nstudies=, iterations=);

data pm;

set &data;

by simruns;

if tau_square_pm=**.** then tau_square_pm=&start_tau_square_pm;

%let max_fin_f_tau=.;

%let _i=0;

%put &max_fin_f_tau;

%put &_i;

*starting the loop;

%do %until(&max_fin_f_tau<=**0** or &_i=&iterations);

%let _i = %eval(&_i+1);

run;

/*proc print data=pm;*/

*calculate weigths and terms that are necessary to calculate the Paule and Mandel estimating equation;

data pm;

set pm;

by simruns;

* Initialize terms that are summed up;

if first.simruns then do;

sum_w_pm=**0**; sum_num_yw_tau=**0**;

end;

* Calculate terms that are summed across studies ...;

w_pm=**1**/(tau_square_pm+vari);

num_yw_tau=w_pm*test_LogOR;

sum_w_pm+w_pm;

sum_num_yw_tau+num_yw_tau;

* and sum them...;

if last.simruns then do;

yw_tau=sum_num_yw_tau/sum_w_pm;

end;

run;

*imput missing column values necessary for further summed terms...;

data pm;

do until(yw_tau);

set pm end=last;

if not missing(yw_tau) then complet_yw_tau=yw_tau;

end;

do until(yw_tau);

set pm end=_last;

output;

end;

run;

/*proc print data=pm;run;*/

*calulate the Paule and Mandel estimating equation (f_tau), the target equation (fin_f_tau) and the correction (delta_tau);

data pm;

set pm;

by simruns;

* Initialize terms that are summed up;

if first.simruns then do;

sum_f_tau=**0**;

sum_deno_delta_tau=**0**;

end;

f_tau=w_pm*(test_LogOR-complet_yw_tau)****2**;

deno_delta_tau=w_pm****2***(test_LogOR-complet_yw_tau)****2**;

* Calculate terms that are summed across studies ...;

sum_f_tau+f_tau;

sum_deno_delta_tau+deno_delta_tau;

* and sum them...;

if last.simruns then do;

a_f_tau=sum_f_tau-(&nstudies-**1**);

fin_f_tau=max(a_f_tau,**0**);

delta_tau=max(**0**, (sum_f_tau-(&nstudies-**1**))/sum_deno_delta_tau);

end;

run;

*determine the maximum of fin_f_tau across meta-analysis so that the loop is repeated until the stoping thresehold is reached in all meta-analysis;

ods select none;

proc means data=pm n;

var study;

output out=outmeanspm max(fin_f_tau)=max_fin_f_tau;

run;

ods select all;

*assign the actuall max_fin_f_tau as macro variable;

data pmeins;

set outmeanspm;

call symputx('max_fin_f_tau', max_fin_f_tau);

run;

%put &max_fin_f_tau;

run;

data pm;

set pm;

by simruns;

* Initialize terms that are summed up;

if first.simruns then do;

sum_tau_square_pm_temp1=**0**;

end;

tau_square_pm_temp1=tau_square_pm+delta_tau;

* Calculate terms that are summed across studies ...;

sum_tau_square_pm_temp1+tau_square_pm_temp1;

* and sum them...;

if last.simruns then do;

tau_square_pm_temp1=sum_tau_square_pm_temp1;

end;

run;

*imput missing column values of tau_square_pm which are necessary for summed terms in the next loop;

data pm;

do until(tau_square_pm_temp1 or last);

set pm end=last;

if not missing(tau_square_pm_temp1) then tau_square_pm_neu=tau_square_pm_temp1;

end;

do until(tau_square_pm_temp1 or _last);

set pm end=_last;

output;

end;

run;

*drop variables that depend on tau_square_pm and conseqeuently change values in each loop;

data pm;

set pm;

by simruns;

tau_square_pm=tau_square_pm_neu;

drop yw_tau sum_w_pm sum_num_yw_tau w_pm num_yw_tau complet_yw_tau sum_f_tau sum_deno_delta_tau f_tau deno_delta_tau a_f_tau fin_f_tau delta_tau sum_tau_square_pm_temp1 tau_square_pm_temp1 tau_square_pm_neu;

run;

/*proc print data=pm;run;*/

%end;

**%mend** PmEstimator;

%***PmEstimator***(data=pmstart, start_tau_square_pm=**0.00001**, nstudies=nstudies, iterations=**100**);

*prepare data for the Paule and Mandel based REM;

**data** rempmeins;

set pm;

by simruns;

* Compute the random effects weight for each study;

rem_w_pm=**1**/(vari+tau_square_pm);

**run**;

* Compute the random effects estimator theta_rem and its variance;

**data** rempm(keep=simruns nstudies LogOR_IVRE_PM rem_w_pm var_theta_rem_pm se_theta_rem_pm tau_square_pm);

set rempmeins end=lastrecord;

by simruns;

* Initialize terms that are summed up;

if first.simruns then do;

sum_rem_times_w_pm=**0**;sum_rem_w_pm=**0**;

end;

* Calculate terms that are summed across studies ...;

test_LogOR_times_rem_w_pm = rem_w_pm*test_LogOR;

* ... and sum them;

sum_rem_w_pm+rem_w_pm;

sum_rem_times_w_pm + test_LogOR_times_rem_w_pm;

* When the last observation is reached, calculate estimates and output them;

if last.simruns then do;

LogOR_IVRE_PM=sum_rem_times_w_pm/sum_rem_w_pm;

var_theta_rem_pm=**1**/sum_rem_w_pm;

se_theta_rem_pm=**1**/sqrt(sum_rem_w_pm);

output;

end;

title"rempm";

**run**;

* ... explode the data set rempm...;

**data** rempmexplode; set rempm; by simruns; do study=**1** to nstudies; output; end; **run**;

* ... and merge with the data set rempmeins;

**data** pmfinal;

merge rempmeins(keep=simruns study test_LogOR rem_w_pm) rempmexplode(keep=simruns study nstudies LogOR_IVRE_PM var_theta_rem_pm

se_theta_rem_pm tau_square_pm);

by simruns study;

**run**;

/*proc print data=pmfinal;run;*/

*Compute the confidence interval adjustment term q;

**data** LogOR_HKPMtemp(keep=simruns nstudies LogOR_IVRE_PM se_theta_rem_pm tau_square_pm q);

set pmfinal end=lastrecord;

by simruns;

* Initialize terms that are summed up;

if first.simruns then do;

sum_num_HKPM_=**0**;

end;

* Calculate Terms that are summed across studies ...;

num_HKPM_ = rem_w_pm*(test_LogOR-LogOR_IVRE_PM)****2**;

* ... and sum them;

sum_num_HKPM_+num_HKPM_;

* When the last observation is reached calculate estimates and output them;

if last.simruns then do;

q=sum_num_HKPM_/(nstudies-**1**);

output;

end;

**run**;

****Knapp-Hartung-Verfahren ohne Varianzkorrektur*********************************************************************;

*calculate confidence intervals;

**data** LogOR_HKPM_(keep=simruns nstudies LogOR_HKPM_ CI95L_LogOR_HKPM_ CI95U_LogOR_HKPM_ tau_square_pm q);

set LogOR_HKPMtemp;

LogOR_HKPM_= LogOR_IVRE_PM;

by simruns;

CI95L_LogOR_HKPM_ = LogOR_HKPM_ - quantile('T',**.975**,nstudies-**1**)* sqrt(q) *se_theta_rem_pm;

CI95U_LogOR_HKPM_ = LogOR_HKPM_ + quantile('T',**.975**,nstudies-**1**)* sqrt(q) *se_theta_rem_pm;

**run**;

/*proc print data=LogOR_HKPM_;title"Hartung/Knapp (PM) ohne Varianzkorrektur"; run;*/

****Knapp-Hartung-Verfahren mit Varianzkorrektur**********************************************************************;

**data** LogOR_MHKPM(keep=simruns nstudies LogOR_MHKPM CI95L_LogOR_MHKPM CI95U_LogOR_MHKPM);

set LogOR_HKPMtemp;

LogOR_MHKPM=LogOR_IVRE_PM;

by simruns;

CI95L_LogOR_MHKPM = LogOR_MHKPM - quantile('T',**.975**,nstudies-**1**)* se_theta_rem_pm;

CI95U_LogOR_MHKPM = LogOR_MHKPM + quantile('T',**.975**,nstudies-**1**)* se_theta_rem_pm;

**run**;

/*proc print data=LogOR_MHKPM;title"Hartung/Knapp (PM) mit Varianzkorrektur"; run;*/

****DerSimonian-Laird-Verfahren*********************************************************************************************;

**data** dl;

set simdatacontinuity;

by simruns;

wsquare_test_LogOR=weight_test_LogOR*weight_test_LogOR;

vari=**1**/weight_test_LogOR;

**run**;

* Compute the heterogeneity statistic Q and the heterogeneity estimator Tau-square;

ods select none;

**proc** **glm** data=dl outstat=outglm(keep=ss _source_ simruns);

model test_LogOR=int / inverse clparm noint;

weight weight_test_LogOR;

by simruns;

title"Estimate Q by PROC GLM";

**run**;

ods select all;

* Determine the number of studies, the sum of weights and the sum of squared weights;

ods select none;

**proc** **means** data=dl n;

var weight_test_LogOR wsquare_test_LogOR;

by simruns;

output out=outmeans n(true_OR)=nstudies sum(weight_test_LogOR)=sumw sum(wsquare_test_LogOR)=sumwsquare;

**run**;

ods select all;

* Compute Q and Tau-square;

**data** qcalcdl(keep=simruns nstudies tau_square_dl Q_dl isquare_dl);

merge outmeans outglm(where=(_SOURCE_="ERROR"));

by simruns;

tau_square_dl=max(**0**,(SS-(nstudies-**1**))/(sumw-sumwsquare/sumw));

isquare_dl=(SS-((nstudies-**1**)))/SS;

Q_dl=SS;

if isquare_dl<**0** then isquare_dl=**0**;

title"Compute Q and Tau-square"; **run**;

* ... explode the data set qcalc ...;

**data** qcalcdlexplode; set qcalcdl; do study=**1** to nstudies; output; end; **run**;

* ... and merge with the original data set;

**data** remeins;

merge dl qcalcdlexplode;

by simruns study;

* Compute the random effects weight for each study;

rem_w=**1**/(vari+tau_square_dl);

**run**;

* Compute the standard random effects estimator theta_rem and its variance;

**data** rem(keep=simruns nstudies LogOR_DLRE_ var_theta_rem se_theta_rem tau_square_DL Q_DL isquare_DL);

set remeins end=lastrecord;

by simruns;

* Initialize terms that are summed up;

if first.simruns then do;

sum_rem_times_w=**0**;sum_rem_w=**0**;

end;

* Calculate terms that are summed across studies ...;

test_LogOR_times_rem_w = rem_w*test_LogOR;

* ... and sum them;

sum_rem_w+rem_w;

sum_rem_times_w + test_LogOR_times_rem_w;

* When the last observation is reached, calculate estimates and output them;

if last.simruns then do;

LogOR_DLRE_=sum_rem_times_w/sum_rem_w;

var_theta_rem=**1**/sum_rem_w;

se_theta_rem=**1**/sqrt(sum_rem_w);

output;

end;

title"rem";

**run**;

/*proc print data=rem;run;*/

* ... explode the data set rem ...;

**data** remexplode; set rem; by simruns; do study=**1** to nstudies; output; end; **run**;

* ... and merge with the data set remeins;

**data** dlre_temp;

merge remeins remexplode;

by simruns study;

**run**;

*Compute the estimator of DerSimonian and Laird;

**data** LogOR_DLRE_(keep=simruns nstudies LogOR_DLRE_ CI95L_LogOR_DLRE_ CI95U_LogOR_DLRE_ tau_square_dl Q_dl isquare_dl);

set dlre_temp end=lastrecord;

by simruns;

* Initialize terms that are summed up;

if first.simruns then do;

sum_rem_w=**0**;

end;

* ... and sum weigths;

sum_rem_w+rem_w;

* When the last observation is reached calculate estimates and output them;

if last.simruns then do;

var_DLRE_=**1**/sum_rem_w;

se_DLRE_=sqrt(var_DLRE_);

CI95L_LogOR_DLRE_ = LogOR_DLRE_ - quantile('normal',**.975**)* se_DLRE_;

CI95U_LogOR_DLRE_ = LogOR_DLRE_ + quantile('normal',**.975**)* se_DLRE_;

output;

end;

title"DerSimonian and Laird";

**run**;

/*proc print data=LogOR_DLRE_;run;*/

*Mantel-Haenszel-Verfahren;

ods select none;

**proc** **freq** data=simdataexplode order=formatted;

tables study*treatment*event / cmh binomialc;

by simruns;

ods output CommonRelRisks=OR_MAHA__temp(where=(StudyType="Fall-Kontroll")

rename=(Value=OR_MAHA_ LowerCL=CI95L_OR_MAHA_ UpperCL=CI95U_OR_MAHA_))

;

format treatment treatf. event eventf.;

title"Mantel-Haenszel analysis";

**run**;

ods select all;

*calculate the stratified Log Odds-Ratio with 95%-CIs and the standard error of the stratified Log Odds-Ratio;

**data** LogOR_MAHA_(keep=simruns LogOR_MAHA_ CI95L_LogOR_MAHA_ CI95U_LogOR_MAHA_);

set OR_MAHA__temp;

LogOR_MAHA_=log(OR_MaHa_); CI95L_LogOR_MAHA_=log(CI95L_OR_MAHA_); CI95U_LogOR_MAHA_=log(CI95U_OR_MAHA_);

**run**;

/*proc print data=OR_MAHA_;run;*/

*Peto-Method-Verfahren;

**data** LogOR_PETO_(keep=simruns LogOR_PETO_ CI95L_LogOR_PETO_ CI95U_LogOR_PETO_);

set simdata end=lastrecord;

by simruns;

* Initialize terms;

if first.simruns then do;

sum_PETO_den=**0**; sum_PETO_num=**0**;

end;

* Calculate Terms that are summed across studies ...;

PETO_den = nt*nc*(st+sc)*(nt+nc-(st+sc))/(((nt+nc)****2**)*(nt+nc-**1**));

PETO_num = (st-(nt*(st+sc)/(nt+nc)));

* ... and sum them;

sum_PETO_den+PETO_den;

sum_PETO_num+PETO_num;

* When the last observation is reached calculate estimates and output them;

if last.simruns then do;

* Standard method;

LogOR_PETO_ = sum_PETO_num/sum_PETO_den;

CI95L_LogOR_PETO_ = LogOR_PETO_ - (probit(**0.975**)/sqrt(sum_PETO_den));

CI95U_LogOR_PETO_ = LogOR_PETO_ + (probit(**0.975**)/sqrt(sum_PETO_den));

output;

end;

title"Peto method";

**run**;

/*proc print data=PETO_;run;*/

*Aggregierte Vierfeldertafel;

ods select none;

**proc** **freq** data=simdataexplode order=formatted;

tables simruns*treatment*event / cmh binomialc;

by simruns;

ods output CommonRelRisks=OR_COLLA_temp(where=(StudyType=" (Odds Ratio)")

rename=(Value=OR_COLLA LowerCL=CI95L_OR_COLLA UpperCL=CI95U_OR_COLLA))

;

format treatment treatf. event eventf.;

title"Collapsed table";

**run**;

ods select all;

*calculate the stratified Log Odds-Ratio with 95%-CIs and the standard error of the stratified Log Odds-Ratio;

**data** LogOR_COLLA(keep=simruns LogOR_COLLA CI95L_LogOR_COLLA CI95U_LogOR_COLLA);

set OR_COLLA_temp;

LogOR_COLLA=log(OR_COLLA); CI95L_LogOR_COLLA=log(CI95L_OR_COLLA); CI95U_LogOR_COLLA=log(CI95U_OR_COLLA);

**run**;

/*proc print data=OR_COLLA_temp;run;*/

*****************************************************************************************************************************************************************************;

*****************************************************************************************************************************************************************************;

********** SUMMARIZE SIMULATION RESULTS *************************************************************************************************************************************;

*****************************************************************************************************************************************************************************;

*****************************************************************************************************************************************************************************;

**data** result_&ident;

* Collect information for this simulation run;

Ident="&ident";

* Merge result data sets from the different procedures;

merge simdata(keep=simruns true_pc true_LogOR true_OR true_TauSquare study nt st nc sc pt pc)

simdatacontinuity(keep=simruns test_OR weight_test_LogOR) LogOR_BBST1 LogOR_BBST2 LogOR_BBFR1

LogOR_BBFR2 LogOR_BBGU1 LogOR_BBGU2 LogOR_BBCB1 LogOR_BBCB2 LogOR_GLFR_ LogOR_GLRRI LogOR_HKPM_

LogOR_MHKPM LogOR_DLRE_ LogOR_MAHA_ LogOR_PETO_ LogOR_COLLA

;

by simruns;

* Macro to calculate performance measures;

**%macro** calcresp(method,true,nullvalue);

bias_&method=&method-true_&true;

mpe_&method=(&method-true_&true)/true_&true;

mse_&method=(&method-true_&true)****2**;

coverage_&method=**.**;

if CI95L_&method<true_&true and CI95U_&method>true_&true then coverage_&method=**1**;

if CI95L_&method>true_&true or CI95U_&method<true_&true then coverage_&method=**0**;

if &method=**.** or CI95L_&method=**.** or CI95U_&method=**.** then coverage_&method=**.**;

ci_length_&method=abs(CI95U_&method-CI95L_&method);

power_&method=**.**;

if CI95L_&method>&nullvalue or CI95U_&method<&nullvalue then power_&method=**1**;

if CI95L_&method<&nullvalue and CI95U_&method>&nullvalue then power_&method=**0**;

if &method=**.** or CI95L_&method=**.** or CI95U_&method=**.** then power_&method=**.**;

**%mend** calcresp;

%***calcresp***(LogOR_BBST1,LogOR,**0**);

%***calcresp***(LogOR_BBST2,LogOR,**0**);

%***calcresp***(LogOR_BBFR1,LogOR,**0**);

%***calcresp***(LogOR_BBFR2,LogOR,**0**);

%***calcresp***(LogOR_BBGU1,LogOR,**0**);

%***calcresp***(LogOR_BBGU2,LogOR,**0**);

%***calcresp***(LogOR_BBCB1,LogOR,**0**);

%***calcresp***(LogOR_BBCB2,LogOR,**0**);

%***calcresp***(LogOR_GLFR_,LogOR,**0**);

%***calcresp***(LogOR_GLRRI,LogOR,**0**);

%***calcresp***(LogOR_HKPM_,LogOR,**0**);

%***calcresp***(LogOR_MHKPM,LogOR,**0**);

%***calcresp***(LogOR_DLRE_,LogOR,**0**);

%***calcresp***(LogOR_MAHA_,LogOR,**0**);

%***calcresp***(LogOR_PETO_,LogOR,**0**);

%***calcresp***(LogOR_COLLA,LogOR,**0**);

**run**;

*Delete data sets;

**proc** **datasets**;

delete Simdata ident simdatadouble simdatahelp simdataquadruple simdataexplode SimpleStats_pt SimpleStats_pc SimpleStats_OR

startingvalues_Beta_Binomial LogOR_BBST_temp LogOR_BBST1 LogOR_BBST2 LogOR_BBFR_temp LogOR_BBFR1 LogOR_BBFR2 LogOR_BBGU_temp

LogOR_BBGU1 LogOR_BBGU2 LogOR_BBCB_temp LogOR_BBCB1 LogOR_BBCB2 LogOR_LogOR_GLFR_ LogOR_GLRRI

pmivre pmstart pm pmeins rempmeins rempm rempmexplode pmfinal LogOR_HKPM_ LogOR_MHKPM dl qcalcdlexplode remeins rem LogOR_DLRE_

LogOR_MAHA_ LogOR_PETO_ LogOR_COLLA;

**quit**;

**%mend** FewStudiesSim2;

*****************************************************************************************************************************************************************************;

*****************************************************************************************************************************************************************************;

********** RUN THE SIMULATION ***********************************************************************************************************************************************;

*****************************************************************************************************************************************************************************;

*****************************************************************************************************************************************************************************;

/*options mlogic mprint;*/

%***FewStudiesSim2***(nstudy=**5**, effect=**0**, rem=**1**, seedsim=**0005**, nsimruns=**10000**, printsimdata=**0**);

**proc** **print** data=result_h0rem05; **run**;

libname results "C:\Users\tmathes\Desktop\SAS";

**data** results.result_h0rem05;

set result_h0rem05;

**run**;

/*%FewStudiesSim2(nstudy=2, effect=1, rem=1, seedsim=0002, nsimruns=10000, printsimdata=0);*/

/*%FewStudiesSim2(nstudy=3, effect=1, rem=1, seedsim=0003, nsimruns=10000, printsimdata=0);*/

/*%FewStudiesSim2(nstudy=4, effect=1, rem=1, seedsim=0004, nsimruns=10000, printsimdata=0);*/

/*%FewStudiesSim2(nstudy=5, effect=1, rem=1, seedsim=0005, nsimruns=10000, printsimdata=0);*/

/*%FewStudiesSim2(nstudy=10, effect=1, rem=1, seedsim=0010, nsimruns=10000, printsimdata=0);*/

/*%FewStudiesSim2(nstudy=2, effect=0, rem=1, seedsim=0002, nsimruns=10000, printsimdata=0);*/

/*%FewStudiesSim2(nstudy=3, effect=0, rem=1, seedsim=0003, nsimruns=10000, printsimdata=0);*/

/*%FewStudiesSim2(nstudy=4, effect=0, rem=1, seedsim=0004, nsimruns=10000, printsimdata=0);*/

/*%FewStudiesSim2(nstudy=5, effect=0, rem=1, seedsim=0005, nsimruns=10000, printsimdata=0);*/

/*%FewStudiesSim2(nstudy=10, effect=0, rem=1, seedsim=0010, nsimruns=10000, printsimdata=0);*/

| **Summary of results** |
| --- |

****************OR*********************************;

***************************************************;

***Complete data se H0*****************************;

libname results "C:\Users\tmathes\Desktop\SAS\OR_H0";

**data** eins_h0;

set results.result_h0rem02 results.result_h0rem03 results.result_h0rem04 results.result_h0rem05 results.result_h0rem10;

**run**;

**data** eins_h0;

set eins_h0 end=lastrecord;

by ident simruns;

if first.simruns then do;

sum_nc=**0**;

end;

sum_nc+nc;

**run**;

**data** eins_h0;

set eins_h0 end=lastrecord;

by ident simruns;

if first.simruns then do;

sum_nt=**0**;

end;

sum_nt+nt;

**run**;

**data** eins_h0;

set eins_h0;

LogOR_CHKPM=LogOR_MHKPM;

if(CI95L_LogOR_DLRE_ > CI95L_LogOR_HKPM_) then do; CI95L_LogOR_CHKPM=CI95L_LogOR_HKPM_; CI95U_LogOR_CHKPM=CI95U_LogOR_HKPM_; bias_LogOR_CHKPM_=bias_LogOR_HKPM_; mse_LogOR_CHKPM=mse_LogOR_HKPM_;

mpe_LogOR_CHKPM=mpe_LogOR_HKPM_; coverage_LogOR_CHKPM=coverage_LogOR_HKPM_; ci_length_LogOR_CHKPM=ci_length_LogOR_HKPM_; power_LogOR_CHKPM=power_LogOR_HKPM_; end;

if(CI95L_LogOR_DLRE_ <= CI95L_LogOR_HKPM_) then do; CI95L_LogOR_CHKPM=CI95L_LogOR_MHKPM; CI95U_LogOR_CHKPM=CI95U_LogOR_MHKPM; bias_LogOR_CHKPM=bias_LogOR_MHKPM; mse_LogOR_CHKPM=mse_LogOR_MHKPM;

mpe_LogOR_CHKPM=mpe_LogOR_MHKPM; coverage_LogOR_CHKPM=coverage_LogOR_MHKPM; ci_length_LogOR_CHKPM=ci_length_LogOR_MHKPM; power_LogOR_CHKPM=power_LogOR_MHKPM; end;

if CI95L_LogOR_BBST1=**.** then do; LogOR_BBST1=**.**; coverage_LogOR_BBST1=**.**; power_LogOR_BBST1=**.**; end;

**run**;

**data** eins_h0;

set eins_h0;

where ((Ident="H0REM02" AND study=**2**) OR (Ident="H0REM03" AND study=**3**) OR (Ident="H0REM04" AND study=**4**) OR (Ident="H0REM05" AND study=**5**) OR (Ident="H0REM05" AND study=**5**) OR (Ident="H0REM10" AND study=**10**));

**run**;

**proc** **contents** data=eins_h0 position;**run**;

**proc** **sort** data=eins_h0;by ident simruns;**run**;

**data** results.gesamt_FEW_STUDIES_2_H0_REM_OR;set eins_h0;**run**;

***Sub-data set H0****************************;

**Heterogenity not significant;

**data** eins_h0_Q_test_temp;

set eins_h0;

p_Q_test=1-probchi(Q_dl,(study-**1**));

**run**;

**data** eins_h0_Q_test;

set eins_h0_Q_test_temp;

where p_Q_test>**0.05**;

**run**;

**proc** **contents** data=eins_h0_Q_test position;**run**;

**proc** **sort** data=eins_h0_Q_test;by ident simruns;**run**;

**data** results.gesamt_FEW_STUDIES_2_H0_REM_OR_Q;set eins_h0_Q_test;**run**;

**at least 200 patients in meta-analysis;

**data** eins_h0_large;

set eins_h0;

where sum_nt>**200**;

**run**;

**proc** **contents** data=eins_h0_large position;**run**;

**proc** **sort** data=eins_h0_large;by ident simruns;**run**;

**data** results.gesamt_FEW_STUDIES_2_H0_REM_OR_L;set eins_h0_large;**run**;

***************************************************

***Complete data set H1****************************;

libname results "C:\Users\tmathes\Desktop\SAS\OR_H1";

**data** eins_h1;

set results.result_h1rem02 results.result_h1rem03 results.result_h1rem04 results.result_h1rem05 results.result_h1rem10;

**run**;

**data** eins_h1;

set eins_h1 end=lastrecord;

by ident simruns;

if first.simruns then do;

sum_nc=**0**;

end;

sum_nc+nc;

**run**;

**data** eins_h1;

set eins_h1 end=lastrecord;

by ident simruns;

if first.simruns then do;

sum_nt=**0**;

end;

sum_nt+nt;

**run**;

**data** eins_h1;

set eins_h1;

LogOR_CHKPM=LogOR_MHKPM;

if(CI95L_LogOR_DLRE_ > CI95L_LogOR_HKPM_) then do; CI95L_LogOR_CHKPM=CI95L_LogOR_HKPM_; CI95U_LogOR_CHKPM=CI95U_LogOR_HKPM_; bias_LogOR_CHKPM_=bias_LogOR_HKPM_; mse_LogOR_CHKPM=mse_LogOR_HKPM_;

mpe_LogOR_CHKPM=mpe_LogOR_HKPM_; coverage_LogOR_CHKPM=coverage_LogOR_HKPM_; ci_length_LogOR_CHKPM=ci_length_LogOR_HKPM_; power_LogOR_CHKPM=power_LogOR_HKPM_; end;

if(CI95L_LogOR_DLRE_ <= CI95L_LogOR_HKPM_) then do; CI95L_LogOR_CHKPM=CI95L_LogOR_MHKPM; CI95U_LogOR_CHKPM=CI95U_LogOR_MHKPM; bias_LogOR_CHKPM=bias_LogOR_MHKPM; mse_LogOR_CHKPM=mse_LogOR_MHKPM;

mpe_LogOR_CHKPM=mpe_LogOR_MHKPM; coverage_LogOR_CHKPM=coverage_LogOR_MHKPM; ci_length_LogOR_CHKPM=ci_length_LogOR_MHKPM; power_LogOR_CHKPM=power_LogOR_MHKPM; end;

if CI95L_LogOR_BBST1=**.** then do; LogOR_BBST1=**.**; coverage_LogOR_BBST1=**.**; power_LogOR_BBST1=**.**; end;

**run**;

**data** eins_h1;

set eins_h1;

where ((Ident="H1REM02" AND study=**2**) OR (Ident="H1REM03" AND study=**3**) OR (Ident="H1REM04" AND study=**4**) OR (Ident="H1REM05" AND study=**5**) OR (Ident="H1REM05" AND study=**5**) OR (Ident="H1REM10" AND study=**10**));

**run**;

**proc** **contents** data=eins_h1 position;**run**;

**proc** **sort** data=eins_h1;by ident simruns;**run**;

**data** results.gesamt_FEW_STUDIES_2_H1_REM_OR;set eins_h1;**run**;

***Sub-data set H1****************************;

**Heterogenity not significant;

**data** eins_h1_Q_test_temp;

set eins_h1;

p_Q_test=1-probchi(Q_dl,(study-**1**));

**run**;

**data** eins_h1_Q_test;

set eins_h1_Q_test_temp;

where p_Q_test>**0.05**;

**run**;

**proc** **contents** data=eins_h1_Q_test position;**run**;

**proc** **sort** data=eins_h1_Q_test;by ident simruns;**run**;

**data** results.gesamt_FEW_STUDIES_2_H1_REM_OR_Q;set eins_h1_Q_test;**run**;

**at least 200 patients in meta-analysis;

**data** eins_h1_large;

set eins_h1;

where sum_nt>**200**;

**run**;

**proc** **contents** data=eins_h1_large position;**run**;

**proc** **sort** data=eins_h1_large;by ident simruns;**run**;

**data** results.gesamt_FEW_STUDIES_2_H1_REM_OR_L;set eins_h1_large;**run**;

****************RR*********************************;

***************************************************;

***Complete data se H0*****************************;

libname results "C:\Users\tmathes\Desktop\SAS\RR_H0";

**data** eins_h0;

set results.result_h0rem02 results.result_h0rem03 results.result_h0rem04 results.result_h0rem05 results.result_h0rem10;

**run**;

**data** eins_h0;

set eins_h0 end=lastrecord;

by ident simruns;

if first.simruns then do;

sum_nc=**0**;

end;

sum_nc+nc;

**run**;

**data** eins_h0;

set eins_h0 end=lastrecord;

by ident simruns;

if first.simruns then do;

sum_nt=**0**;

end;

sum_nt+nt;

**run**;

**data** eins_h0;

set eins_h0;

LogRR_CHKPM=LogRR_MHKPM;

if(CI95L_LogRR_DLRE_ > CI95L_LogRR_HKPM_) then do; CI95L_LogRR_CHKPM=CI95L_LogRR_HKPM_; CI95U_LogRR_CHKPM=CI95U_LogRR_HKPM_; bias_LogRR_CHKPM_=bias_LogRR_HKPM_; mse_LogRR_CHKPM=mse_LogRR_HKPM_;

mpe_LogRR_CHKPM=mpe_LogRR_HKPM_; coverage_LogRR_CHKPM=coverage_LogRR_HKPM_; ci_length_LogRR_CHKPM=ci_length_LogRR_HKPM_; power_LogRR_CHKPM=power_LogRR_HKPM_; end;

if(CI95L_LogRR_DLRE_ <= CI95L_LogRR_HKPM_) then do; CI95L_LogRR_CHKPM=CI95L_LogRR_MHKPM; CI95U_LogRR_CHKPM=CI95U_LogRR_MHKPM; bias_LogRR_CHKPM=bias_LogRR_MHKPM; mse_LogRR_CHKPM=mse_LogRR_MHKPM;

mpe_LogRR_CHKPM=mpe_LogRR_MHKPM; coverage_LogRR_CHKPM=coverage_LogRR_MHKPM; ci_length_LogRR_CHKPM=ci_length_LogRR_MHKPM; power_LogRR_CHKPM=power_LogRR_MHKPM; end;

if CI95L_LogRR_BBST1=**.** then do; LogRR_BBST1=**.**; coverage_LogRR_BBST1=**.**; power_LogRR_BBST1=**.**; end;

**run**;

**data** eins_h0;

set eins_h0;

where ((Ident="H0REM02" AND study=**2**) OR (Ident="H0REM03" AND study=**3**) OR (Ident="H0REM04" AND study=**4**) OR (Ident="H0REM05" AND study=**5**) OR (Ident="H0REM05" AND study=**5**) OR (Ident="H0REM10" AND study=**10**));

**run**;

**proc** **contents** data=eins_h0 position;**run**;

**proc** **sort** data=eins_h0;by ident simruns;**run**;

**data** results.gesamt_FEW_STUDIES_2_H0_REM_RR;set eins_h0;**run**;

***Sub-data set H0****************************;

**Heterogenity not significant;

**data** eins_h0_Q_test_temp;

set eins_h0;

p_Q_test=1-probchi(Q_dl,(study-**1**));

**run**;

**data** eins_h0_Q_test;

set eins_h0_Q_test_temp;

where p_Q_test>**0.05**;

**run**;

**proc** **contents** data=eins_h0_Q_test position;**run**;

**proc** **sort** data=eins_h0_Q_test;by ident simruns;**run**;

**data** results.gesamt_FEW_STUDIES_2_H0_REM_RR_Q;set eins_h0_Q_test;**run**;

**at least 200 patients in meta-analysis;

**data** eins_h0_large;

set eins_h0;

where sum_nt>**200**;

**run**;

**proc** **contents** data=eins_h0_large position;**run**;

**proc** **sort** data=eins_h0_large;by ident simruns;**run**;

**data** results.gesamt_FEW_STUDIES_2_H0_REM_RR_L;set eins_h0_large;**run**;

***************************************************

***Complete data set H1****************************;

libname results "C:\Users\tmathes\Desktop\SAS\RR_H1";

**data** eins_h1;

set results.result_h1rem02 results.result_h1rem03 results.result_h1rem04 results.result_h1rem05 results.result_h1rem10;

**run**;

**data** eins_h1;

set eins_h1 end=lastrecord;

by ident simruns;

if first.simruns then do;

sum_nc=**0**;

end;

sum_nc+nc;

**run**;

**data** eins_h1;

set eins_h1 end=lastrecord;

by ident simruns;

if first.simruns then do;

sum_nt=**0**;

end;

sum_nt+nt;

**run**;

**data** eins_h1;

set eins_h1;

LogRR_CHKPM=LogRR_MHKPM;

if(CI95L_LogRR_DLRE_ > CI95L_LogRR_HKPM_) then do; CI95L_LogRR_CHKPM=CI95L_LogRR_HKPM_; CI95U_LogRR_CHKPM=CI95U_LogRR_HKPM_; bias_LogRR_CHKPM_=bias_LogRR_HKPM_; mse_LogRR_CHKPM=mse_LogRR_HKPM_;

mpe_LogRR_CHKPM=mpe_LogRR_HKPM_; coverage_LogRR_CHKPM=coverage_LogRR_HKPM_; ci_length_LogRR_CHKPM=ci_length_LogRR_HKPM_; power_LogRR_CHKPM=power_LogRR_HKPM_; end;

if(CI95L_LogRR_DLRE_ <= CI95L_LogRR_HKPM_) then do; CI95L_LogRR_CHKPM=CI95L_LogRR_MHKPM; CI95U_LogRR_CHKPM=CI95U_LogRR_MHKPM; bias_LogRR_CHKPM=bias_LogRR_MHKPM; mse_LogRR_CHKPM=mse_LogRR_MHKPM;

mpe_LogRR_CHKPM=mpe_LogRR_MHKPM; coverage_LogRR_CHKPM=coverage_LogRR_MHKPM; ci_length_LogRR_CHKPM=ci_length_LogRR_MHKPM; power_LogRR_CHKPM=power_LogRR_MHKPM; end;

if CI95L_LogRR_BBST1=**.** then do; LogRR_BBST1=**.**; coverage_LogRR_BBST1=**.**; power_LogRR_BBST1=**.**; end;

**run**;

**data** eins_h1;

set eins_h1;

where ((Ident="H1REM02" AND study=**2**) OR (Ident="H1REM03" AND study=**3**) OR (Ident="H1REM04" AND study=**4**) OR (Ident="H1REM05" AND study=**5**) OR (Ident="H1REM05" AND study=**5**) OR (Ident="H1REM10" AND study=**10**));

**run**;

**proc** **contents** data=eins_h1 position;**run**;

**proc** **sort** data=eins_h1;by ident simruns;**run**;

**data** results.gesamt_FEW_STUDIES_2_H1_REM_RR;set eins_h1;**run**;

***Sub-data set H1****************************;

**Heterogenity not significant;

**data** eins_h1_Q_test_temp;

set eins_h1;

p_Q_test=1-probchi(Q_dl,(study-**1**));

**run**;

**data** eins_h1_Q_test;

set eins_h1_Q_test_temp;

where p_Q_test>**0.05**;

**run**;

**proc** **contents** data=eins_h1_Q_test position;**run**;

**proc** **sort** data=eins_h1_Q_test;by ident simruns;**run**;

**data** results.gesamt_FEW_STUDIES_2_H1_REM_RR_Q;set eins_h1_Q_test;**run**;

**at least 200 patients in meta-analysis;

**data** eins_h1_large;

set eins_h1;

where sum_nt>**200**;

**run**;

**proc** **contents** data=eins_h1_large position;**run**;

**proc** **sort** data=eins_h1_large;by ident simruns;**run**;

**data** results.gesamt_FEW_STUDIES_2_H1_REM_RR_L;set eins_h1_large;**run**;
