# Supplementary material for: Performance of several types of beta-binomial models in comparison to standard approaches for meta-analyses with very few studies
Source: BMC Med Res Methodol. 2022 Dec 13;22:319. doi: 10.1186/s12874-022-01779-3 (PMC9745934; doi:10.1186/s12874-022-01779-3)
Supplement: Supplementary file 2 — Additional file 2: Table S1. Number of converged simulation runs (out of 10,000) for the odds ratio under the null hypothesis. Table S2. (Absolute) bias for the log odds ratio under the null hypothesis. Table S3. Coverage probability (%) for the odds ratio under the null hypothesis. Table S4. Length of 95% confidence interval for the log odds ratio under the null hypothesis. Table S5. Number of converged simulation runs (out of 10,000) for the odds ratio under the alternative hypothesis. Table S6. (Absolute) bias for the log odds ratio under the alternative hypothesis. Table S7. Percentage bias (%) for the log odds ratio under the alternative hypothesis. Table S8. Coverage probability (%) for the odds ratio under the alternative hypothesis. Table S9. Length of 95% confidence interval for the log odds ratio under the alternative hypothesis. Table S10. Power (%) for the odds ratio under the alternative hypothesis. Table S11. Number of converged simulation runs (out of 10,000) for the relative risk under the null hypothesis. Table S12. (Absolute) bias for the log relative risk under the null hypothesis. Table S13. Coverage probability (%) for the relative risk under the null hypothesis. Table S14. Length of 95% confidence interval for the log relative risk under the null hypothesis. Table S15. Number of converged simulation runs (out of 10,000) for the relative risk under the alternative hypothesis. Table S16. (Absolute) bias of the log relative risk under the alternative hypothesis. Table S17. Percentage bias (%) for the log relative risk under the alternative hypothesis. Table S18. Coverage probability (%) for the relative risk under the alternative hypothesis. Table S19. Length of 95% confidence interval for the log relative risk under the alternative hypothesis. Table S20. Power (%) for the relative risk under the alternative hypothesis. [file 12874_2022_1779_MOESM2_ESM.docx]

------------------------------------------------------------------------ **Odds Ratio under H_0_** ----------------------------------------------------------------------

Table S1: **Number of converged simulation runs** (out of 10,000) for the odds ratio under the null hypothesis

| **Number of studies** | **BBST** | **BBFR** | **BBCB1** | **BBCB2** | **GLFR** | **GLRRI** | **HKSJ** | **DSL** | **MH** | **POR** | **COLL** |
| --- | --- | --- | --- | --- | --- | --- | --- | --- | --- | --- | --- |
| 2 | 9778 | 9778 | 9603 | 9520 | 9995 | 8469 | 9977 | 9977 | 9394 | 9999 | 9999 |
| 3 | 9825 | 9825 | 9737 | 9700 | 9994 | 7900 | 9993 | 9993 | 9776 | 9999 | 9999 |
| 4 | 9854 | 9854 | 9793 | 9725 | 9994 | 7682 | 9997 | 9997 | 9937 | 10000 | 10000 |
| 5 | 9860 | 9859 | 9843 | 9792 | 9992 | 7445 | 9996 | 9996 | 9977 | 10000 | 10000 |
| 10 | 9909 | 9909 | 9874 | 9823 | 9988 | 6896 | 10000 | 10000 | 10000 | 10000 | 10000 |

Table S2: **(Absolute) bias** for the log odds ratio under the null hypothesis

| **Number of studies** | **BBST** | **BBFR** | **BBCB1** | **BBCB2** | **GLFR** | **GLRRI** | **HKSJ** | **DSL** | **MH** | **POR** | **COLL** |
| --- | --- | --- | --- | --- | --- | --- | --- | --- | --- | --- | --- |
|  | Median  Q1  Q3 | Median  Q1  Q3 | Median  Q1  Q3 | Median  Q1  Q3 | Median  Q1  Q3 | Median  Q1  Q3 | Median  Q1  Q3 | Median  Q1  Q3 | Median  Q1  Q3 | Median  Q1  Q3 | Median  Q1  Q3 |
| 2 | 0.0004  −0.3857  0.3907 | 0.0004  −0.3857  0.3907 | −0.0090  −0.3957  0.3652 | −0.0000  −0.2880  0.2877 | 0.0112  −0.3846  0.4325 | −0.0145  −0.4081  0.3569 | 0.0065  −0.3692  0.3880 | 0.0065  −0.3692  0.3880 | 0.0162  −0.3413  0.4037 | 0.0112  −0.3787  0.4292 | 0.0086  −0.3825  0.4336 |
| 3 | 0.0109  −0.2994  0.3497 | 0.0109  −0.2994  0.3497 | −0.0098  −0.3262  0.3253 | −0.0000  −0.2476  0.2515 | 0.0239  −0.2965  0.3963 | 0.0014  −0.3081  0.3338 | 0.0184  −0.2783  0.3405 | 0.0186  −0.2765  0.3388 | 0.0302  −0.2775  0.3945 | 0.0299  −0.2861  0.4033 | 0.0295  −0.2856  0.4084 |
| 4 | 0.0119  −0.2522  0.2943 | 0.0119  −0.2522  0.2943 | −0.0058  −0.2772  0.2657 | −0.0000  −0.2151  0.2179 | 0.0229  −0.2466  0.3326 | 0.0078  −0.2598  0.3026 | 0.0144  −0.2346  0.2905 | 0.0148  −0.2335  0.2890 | 0.0288  −0.2357  0.3420 | 0.0281  −0.2379  0.3446 | 0.0275  −0.2386  0.3432 |
| 5 | 0.0167  −0.2099  0.2640 | 0.0166  −0.2099  0.2639 | −0.0074  −0.2468  0.2345 | 0.0000  −0.1815  0.1894 | 0.0250  −0.2097  0.3066 | 0.0168  −0.2123  0.2744 | 0.0196  −0.2049  0.2654 | 0.0200  −0.2022  0.2666 | 0.0328  −0.2013  0.3261 | 0.0324  −0.2032  0.3267 | 0.0362  −0.1989  0.3247 |
| 10 | 0.0176  −0.1438  0.1883 | 0.0176  −0.1438  0.1883 | −0.0051  −0.1815  0.1571 | 0.0000  −0.1293  0.1336 | 0.0293  −0.1336  0.2248 | 0.0185  −0.1475  0.1997 | 0.0221  −0.1297  0.1935 | 0.0230  −0.1293  0.1934 | 0.0386  −0.1314  0.2609 | 0.0387  −0.1321  0.2623 | 0.0372  −0.1302  0.2577 |

Q1: 1st quartile; Q3: 3rd quartile

Table S3: **Coverage probability (%)** for the odds ratio under the null hypothesis

| **Number of studies** | **BBST** | | **BBFR** | | **BBCB1** | | **BBCB2** | | **GLFR** | **GLRRI** | **HKSJ** | **DSL** | **MH** | **POR** | **COLL** |
| --- | --- | --- | --- | --- | --- | --- | --- | --- | --- | --- | --- | --- | --- | --- | --- |
|  | K – 1 | 2K – 2 | K – 1 | 2K – 2 | K – 1 | 2K – 2 | K – 1 | 2K – 2 |  |  |  |  |  |  |  |
| 2 | 99.90 | 97.95 | 99.89 | 97.98 | 99.38 | 97.30 | 99.33 | 98.93 | 99.79 | 85.68 | 99.80 | 90.79 | 81.59 | 80.58 | 82.24 |
| 3 | 98.81 | 96.05 | 98.81 | 96.05 | 98.40 | 95.56 | 99.37 | 97.52 | 96.80 | 85.58 | 98.85 | 92.06 | 80.18 | 78.40 | 80.14 |
| 4 | 98.13 | 95.47 | 98.13 | 95.47 | 97.82 | 94.98 | 98.72 | 96.48 | 94.99 | 84.86 | 98.33 | 92.49 | 79.00 | 77.63 | 79.13 |
| 5 | 97.72 | 95.37 | 97.72 | 95.36 | 97.51 | 95.16 | 98.29 | 96.26 | 93.88 | 84.15 | 97.91 | 93.13 | 77.15 | 76.14 | 77.01 |
| 10 | 96.43 | 94.98 | 96.43 | 94.98 | 95.57 | 94.29 | 96.06 | 94.77 | 90.39 | 82.41 | 96.23 | 93.33 | 73.82 | 73.27 | 73.96 |

Table S4: **Length of 95 % confidence interval** for the log odds ratio under the null hypothesis

| **Number of studies** | **BBST** | | **BBFR** | | **BBCB1** | | **BBCB2** | | **GLFR** | **GLRRI** | **HKSJ** | **DSL** | **MH** | **POR** | **COLL** |
| --- | --- | --- | --- | --- | --- | --- | --- | --- | --- | --- | --- | --- | --- | --- | --- |
|  | K – 1 | 2K – 2 | K – 1 | 2K – 2 | K – 1 | 2K – 2 | K – 1 | 2K – 2 |  |  |  |  |  |  |  |
|  | Median  Q1  Q3 | Median  Q1  Q3 | Median  Q1  Q3 | Median  Q1  Q3 | Median  Q1  Q3 | Median  Q1  Q3 | Median  Q1  Q3 | Median  Q1  Q3 | Median  Q1  Q3 | Median  Q1  Q3 | Median  Q1  Q3 | Median  Q1  Q3 | Median  Q1  Q3 | Median  Q1  Q3 | Median  Q1  Q3 |
| 2 | 13.1126  7.8713  23.4222 | 4.4403  2.6654  7.9314 | 13.1126  7.8702  23.4222 | 4.4403  2.6650  7.9314 | 12.9558  7.7496  22.9086 | 4.3872  2.6242  7.7574 | 10.8814  5.9834  20.6388 | 3.6847  2.0261  6.9888 | 12.4947  7.5389  23.3022 | 1.8487  1.1038  3.4538 | 14.1601  7.7049  25.3066 | 2.5465  1.4191  4.2315 | 1.6367  1.0465  2.8414 | 1.6598  1.0549  2.9228 | 1.7256  1.0817  3.2336 |
| 3 | 3.7961  2.2461  6.6363 | 2.4496  1.4494  4.2823 | 3.7961  2.2461  6.6363 | 2.4496  1.4494  4.2823 | 3.8127  2.2471  6.6360 | 2.4603  1.4500  4.2821 | 3.1888  1.7026  5.8710 | 2.0577  1.0987  3.7885 | 3.4106  2.0936  6.3203 | 1.5129  0.8841  2.9144 | 4.3827  2.5054  6.8549 | 2.0281  1.1865  3.2792 | 1.2810  0.8478  2.3537 | 1.2663  0.8402  2.2938 | 1.3049  0.8548  2.4732 |
| 4 | 2.4517  1.4600  4.1386 | 1.8850  1.1225  3.1821 | 2.4517  1.4600  4.1386 | 1.8850  1.1225  3.1821 | 2.4740  1.4619  4.1621 | 1.9022  1.1241  3.2001 | 2.0282  1.1027  3.7137 | 1.5594  0.8478  2.8554 | 2.1652  1.3430  3.8901 | 1.3073  0.7607  2.4804 | 2.8089  1.6347  4.3356 | 1.7153  1.0198  2.7188 | 1.0704  0.7257  1.9908 | 1.0451  0.7192  1.9187 | 1.0702  0.7253  2.0229 |
| 5 | 1.9463  1.1558  3.2439 | 1.6165  0.9599  2.6942 | 1.9459  1.1554  3.2431 | 1.6162  0.9596  2.6936 | 1.9671  1.1622  3.2510 | 1.6338  0.9652  2.7002 | 1.6006  0.8769  2.8674 | 1.3294  0.7283  2.3815 | 1.6974  1.0538  3.0376 | 1.1629  0.6784  2.1689 | 2.1806  1.2838  3.3875 | 1.5158  0.9046  2.3956 | 0.9174  0.6326  1.7122 | 0.9058  0.6295  1.6418 | 0.9161  0.6316  1.7274 |
| 10 | 1.1586  0.6805  1.8740 | 1.0760  0.6320  1.7404 | 1.1586  0.6805  1.8740 | 1.0760  0.6320  1.7404 | 1.1624  0.6839  1.8667 | 1.0796  0.6351  1.7336 | 0.9206  0.5068  1.6590 | 0.8550  0.4707  1.5407 | 0.9788  0.6012  1.7447 | 0.8194  0.4572  1.5174 | 1.2340  0.7326  1.9143 | 1.0463  0.6255  1.6381 | 0.5948  0.4337  1.1510 | 0.5902  0.4346  1.1067 | 0.5932  0.4331  1.1572 |

Q1: 1st quartile; Q3: 3rd quartile

------------------------------------------------------------------------ **Odds Ratio under H_1_** ----------------------------------------------------------------------

Table S5: **Number of converged simulation runs** (out of 10,000) for the odds ratio under the alternative hypothesis

| **Number of Studies** | **BBST** | **BBFR** | **BBCB1** | **BBCB2** | **GLFR** | **GLRRI** | **HKSJ** | **DSL** | **MH** | **POR** | **COLL** |
| --- | --- | --- | --- | --- | --- | --- | --- | --- | --- | --- | --- |
| 2 | 9786 | 9786 | 9538 | 9453 | 9992 | 8590 | 9987 | 9987 | 9151 | 9999 | 9999 |
| 3 | 9831 | 9831 | 9723 | 9659 | 9990 | 8095 | 9989 | 9989 | 9691 | 9998 | 9998 |
| 4 | 9882 | 9882 | 9794 | 9764 | 9990 | 7830 | 9997 | 9997 | 9863 | 9999 | 9999 |
| 5 | 9875 | 9875 | 9827 | 9802 | 9994 | 7646 | 9995 | 9995 | 9927 | 10000 | 10000 |
| 10 | 9943 | 9943 | 9868 | 9838 | 9988 | 7083 | 9999 | 9999 | 9995 | 10000 | 10000 |

Table S6: **(Absolute) bias** for the log odds ratio under the alternative hypothesis

| **Number of studies** | **BBST** | **BBFR** | **BBCB1** | **BBCB2** | **GLFR** | **GLRRI** | **HKSJ** | **DSL** | **MH** | **POR** | **COLL** |
| --- | --- | --- | --- | --- | --- | --- | --- | --- | --- | --- | --- |
|  | Median  Q1  Q3 | Median  Q1  Q3 | Median  Q1  Q3 | Median  Q1  Q3 | Median  Q1  Q3 | Median  Q1  Q3 | Median  Q1  Q3 | Median  Q1  Q3 | Median  Q1  Q3 | Median  Q1  Q3 | Median  Q1  Q3 |
| 2 | 0.0203  −0.4137  0.4385 | 0.0203  −0.4137  0.4385 | 0.0224  −0.3925  0.4287 | 0.1378  −0.2316  0.4747 | 0.0251  −0.4195  0.4758 | −0.0029  −0.4539  0.4033 | 0.0478  −0.3418  0.4699 | 0.0478  −0.3418  0.4699 | 0.0628  −0.3020  0.4782 | 0.0373  −0.3683  0.4755 | 0.0288  −0.4098  0.4764 |
| 3 | 0.0225  −0.3057  0.3762 | 0.0225  −0.3057  0.3762 | 0.0131  −0.3130  0.3609 | 0.1327  −0.1817  0.4300 | 0.0283  −0.3117  0.3990 | 0.0061  −0.3412  0.3590 | 0.0608  −0.2443  0.4080 | 0.0620  −0.2392  0.4067 | 0.0478  −0.2707  0.4138 | 0.0482  −0.2692  0.4140 | 0.0348  −0.3015  0.4119 |
| 4 | 0.0215  −0.2610  0.3136 | 0.0215  −0.2610  0.3136 | 0.0081  −0.2798  0.2917 | 0.1258  −0.1505  0.3761 | 0.0220  −0.2676  0.3335 | 0.0086  −0.2850  0.3071 | 0.0545  −0.2060  0.3499 | 0.0553  −0.2029  0.3516 | 0.0412  −0.2402  0.3644 | 0.0460  −0.2280  0.3636 | 0.0350  −0.2537  0.3588 |
| 5 | 0.0209  −0.2298  0.2830 | 0.0209  −0.2298  0.2830 | 0.0065  −0.2563  0.2595 | 0.1187  −0.1278  0.3606 | 0.0227  −0.2384  0.3057 | 0.0086  −0.2550  0.2714 | 0.0535  −0.1639  0.3232 | 0.0545  −0.1634  0.3252 | 0.0390  −0.2155  0.3348 | 0.0457  −0.1977  0.3358 | 0.0369  −0.2228  0.3292 |
| 10 | 0.0207  −0.1409  0.2119 | 0.0207  −0.1409  0.2119 | 0.0011  −0.1684  0.1779 | 0.1073  −0.0698  0.2858 | 0.0235  −0.1486  0.2244 | 0.0135  −0.1603  0.2045 | 0.0525  −0.0984  0.2556 | 0.0532  −0.0984  0.2582 | 0.0390  −0.1341  0.2668 | 0.0490  −0.1189  0.2724 | 0.0386  −0.1351  0.2660 |

Q1: 1st quartile; Q3: 3rd quartile

Table S7: **Percentage bias (%)** for the log odds ratio under the alternative hypothesis

| **Number of studies** | **BBST** | **BBFR** | **BBCB1** | **BBCB2** | **GLFR** | **GLRRI** | **HKSJ** | **DSL** | **MH** | **POR** | **COLL** |
| --- | --- | --- | --- | --- | --- | --- | --- | --- | --- | --- | --- |
|  | Median  Q1  Q3 | Median  Q1  Q3 | Median  Q1  Q3 | Median  Q1  Q3 | Median  Q1  Q3 | Median  Q1  Q3 | Median  Q1  Q3 | Median  Q1  Q3 | Median  Q1  Q3 | Median  Q1  Q3 | Median  Q1  Q3 |
| 2 | −5.11  −123.48  129.70 | −5.11  −123.48  129.70 | −5.19  −121.31  120.92 | −36.47  −108.22  71.37 | −6.08  −134.08  131.63 | 0.69  −109.32  146.04 | −11.18  −129.21  95.25 | −11.18  −129.21  95.25 | −15.35  −137.58  84.97 | −9.00  −134.09  106.05 | −6.80  −135.76  115.49 |
| 3 | −5.15  −109.29  88.72 | −5.15  −109.29  88.72 | −3.16  −104.89  91.03 | −35.18  −100.79  53.83 | −6.79  −120.90  90.55 | −1.48  −100.34  98.71 | −14.07  −113.60  67.96 | −13.90  −113.90  66.87 | −11.23  −126.35  75.77 | −10.52  −124.50  76.43 | −8.24  −123.08  85.81 |
| 4 | −4.71  −88.62  77.02 | −4.71  −88.62  77.02 | −1.71  −82.00  82.45 | −32.30  −99.91  47.71 | −5.23  −96.81  78.32 | −1.88  −86.00  84.61 | −12.62  −95.54  57.25 | −12.57  −96.22  56.93 | −9.29  −107.39  69.32 | −10.03  −105.76  64.88 | −7.92  −106.22  73.31 |
| 5 | −5.07  −81.65  65.71 | −5.07  −81.65  65.71 | −1.44  −72.16  74.12 | −31.51  −91.35  38.30 | −5.24  −91.15  66.97 | −1.84  −77.00  72.48 | −12.35  −89.98  47.04 | −12.74  −90.59  45.97 | −9.35  −102.20  59.73 | −10.33  −101.29  55.51 | −8.88  −101.09  62.24 |
| 10 | −5.11  −55.87  40.20 | −5.11  −55.87  40.20 | −0.26  c−47.14  48.89 | −28.08  −71.48  20.74 | −5.71  −62.81  41.06 | −3.18  −54.78  44.85 | −12.83  −66.42  27.47 | −12.84  −66.33  27.21 | −8.70  −77.65  36.99 | −10.68  −77.10  32.64 | −9.01  −77.40  37.12 |

Q1: 1st quartile; Q3: 3rd quartile

Table S8: **Coverage probability (%)** for the odds ratio under the alternative hypothesis

| **Number of studies** | **BBST** | | **BBFR** | | **BBCB1** | | **BBCB2** | | **GLFR** | **GLRRI** | **HKSJ** | **DSL** | **MH** | **POR** | **COLL** |
| --- | --- | --- | --- | --- | --- | --- | --- | --- | --- | --- | --- | --- | --- | --- | --- |
|  | K – 1 | 2K – 2 | K – 1 | 2K – 2 | K – 1 | 2K – 2 | K – 1 | 2K – 2 |  |  |  |  |  |  |  |
| 2 | 99.93 | 98.25 | 99.92 | 98.25 | 99.39 | 97.54 | 99.43 | 97.93 | 99.84 | 86.64 | 99.86 | 91.24 | 82.21 | 82.09 | 83.29 |
| 3 | 98.65 | 96.08 | 98.65 | 96.08 | 98.22 | 95.65 | 97.76 | 93.37 | 96.93 | 86.23 | 98.80 | 92.41 | 81.20 | 80.52 | 81.54 |
| 4 | 97.73 | 95.27 | 97.73 | 95.27 | 97.50 | 94.92 | 94.80 | 90.32 | 94.93 | 85.52 | 98.15 | 92.51 | 78.92 | 78.41 | 79.13 |
| 5 | 97.09 | 94.50 | 97.09 | 94.50 | 96.94 | 94.31 | 92.06 | 88.16 | 93.27 | 84.29 | 97.40 | 92.41 | 77.56 | 77.15 | 77.64 |
| 10 | 95.24 | 93.70 | 95.24 | 93.70 | 94.72 | 93.19 | 85.08 | 82.81 | 90.50 | 83.44 | 95.39 | 92.02 | 75.05 | 75.13 | 75.12 |

Table S9: **Length of 95 % confidence interval** for the log odds ratio under the alternative hypothesis

| **Number of studies** | **BBST** | | **BBFR** | | **BBCB1** | | **BBCB2** | | **GLFR** | **GLRRI** | **HKSJ** | **DSL** | **MH** | **POR** | **COLL** |
| --- | --- | --- | --- | --- | --- | --- | --- | --- | --- | --- | --- | --- | --- | --- | --- |
|  | K – 1 | 2K – 2 | K – 1 | 2K – 2 | K – 1 | 2K – 2 | K – 1 | 2K – 2 |  |  |  |  |  |  |  |
|  | Median  Q1  Q3 | Median  Q1  Q3 | Median  Q1  Q3 | Median  Q1  Q3 | Median  Q1  Q3 | Median  Q1  Q3 | Median  Q1  Q3 | Median  Q1  Q3 | Median  Q1  Q3 | Median  Q1  Q3 | Median  Q1  Q3 | Median  Q1  Q3 | Median  Q1  Q3 | Median  Q1  Q3 | Median  Q1  Q3 |
| 2 | 13.9376  8.2246  25.9345 | 4.7196  2.7851  8.7821 | 13.9361  8.2246  25.9345 | 4.7191  2.7851  8.7821 | 13.6915  8.1041  24.3445 | 4.6363  2.7443  8.2437 | 11.7420  6.7764  21.9844 | 3.9761  2.2947  7.4445 | 13.3429  7.9516  26.0939 | 2.0243  1.1683  4.0264 | 14.7509  8.1800  26.2114 | 2.6849  1.4853  4.3020 | 1.6676  1.0936  2.9311 | 1.7179  1.0918  3.1537 | 1.8261  1.1331  3.6004 |
| 3 | 3.9920  2.3861  7.0423 | 2.5760  1.5397  4.5443 | 3.9920  2.3861  7.0423 | 2.5760  1.5397  4.5443 | 3.9786  2.3701  6.9088 | 2.5674  1.5294  4.4582 | 3.4446  1.9509  6.2910 | 2.2228  1.2589  4.0595 | 3.6592  2.2347  6.7651 | 1.6448  0.9573  3.2227 | 4.6455  2.6202  7.0428 | 2.1729  1.2512  3.3399 | 1.3792  0.8928  2.5343 | 1.3435  0.8745  2.4320 | 1.4145  0.9020  2.6915 |
| 4 | 2.5672  1.4981  4.3848 | 1.9739  1.1519  3.3714 | 2.5672  1.4981  4.3848 | 1.9739  1.1519  3.3714 | 2.5609  1.5004  4.3504 | 1.9690  1.1536  3.3449 | 2.1868  1.2270  3.9709 | 1.6814  0.9434  3.0532 | 2.3064  1.3795  4.1754 | 1.4252  0.7942  2.6799 | 2.9237  1.6751  4.4756 | 1.7900  1.0436  2.8363 | 1.1108  0.7362  2.1306 | 1.0772  0.7210  2.0366 | 1.1215  0.7371  2.1993 |
| 5 | 2.0319  1.1976  3.4433 | 1.6876  0.9947  2.8599 | 2.0319  1.1976  3.4433 | 1.6876  0.9947  2.8599 | 2.0471  1.2085  3.4401 | 1.7003  1.0037  2.8572 | 1.7140  0.9899  3.1329 | 1.4236  0.8222  2.6021 | 1.8037  1.0934  3.2947 | 1.2365  0.6983  2.4007 | 2.2968  1.3476  3.5014 | 1.5856  0.9515  2.4901 | 0.9778  0.6596  1.9020 | 0.9432  0.6456  1.7836 | 0.9833  0.6584  1.9269 |
| 10 | 1.1985  0.7009  1.9566 | 1.1130  0.6509  1.8172 | 1.1985  0.7009  1.9566 | 1.1130  0.6509  1.8172 | 1.1958  0.7032  1.9258 | 1.1106  0.6531  1.7885 | 1.0095  0.5956  1.7500 | 0.9375  0.5532  1.6253 | 1.0494  0.6264  1.8467 | 0.8898  0.4901  1.6570 | 1.2806  0.7605  1.9764 | 1.0954  0.6500  1.6887 | 0.6405  0.4504  1.2676 | 0.6233  0.4448  1.2094 | 0.6389  0.4490  1.2682 |

Q1: 1st quartile; Q3: 3rd quartile

Table S10: **Power (%)** for the odds ratio under the alternative hypothesis

| **Number of studies** | **BBST** | | **BBFR** | | **BBCB1** | | **BBCB2** | | **GLFR** | **GLRRI** | **HKSJ** | **DSL** | **MH** | **POR** | **COLL** |
| --- | --- | --- | --- | --- | --- | --- | --- | --- | --- | --- | --- | --- | --- | --- | --- |
|  | K – 1 | 2K – 2 | K – 1 | 2K – 2 | K – 1 | 2K – 2 | K – 1 | 2K – 2 |  |  |  |  |  |  |  |
| 2 | 0.05 | 4.36 | 0.06 | 4.36 | 0.60 | 5.06 | 0.57 | 1.41 | 0.10 | 24.98 | 0.25 | 17.94 | 30.25 | 30.74 | 28.29 |
| 3 | 5.28 | 13.97 | 5.28 | 13.97 | 5.56 | 14.55 | 1.42 | 8.09 | 7.33 | 29.67 | 4.55 | 20.25 | 34.73 | 36.60 | 34.35 |
| 4 | 12.60 | 20.49 | 12.60 | 20.49 | 13.03 | 20.97 | 6.87 | 14.14 | 16.26 | 32.76 | 10.29 | 23.73 | 39.60 | 41.31 | 39.31 |
| 5 | 17.66 | 24.18 | 17.66 | 24.18 | 18.29 | 24.82 | 11.33 | 17.94 | 22.55 | 37.88 | 14.94 | 26.05 | 43.14 | 45.01 | 43.13 |
| 10 | 33.28 | 36.40 | 33.28 | 36.40 | 35.11 | 38.24 | 29.63 | 33.27 | 38.97 | 47.93 | 28.98 | 35.27 | 54.30 | 55.14 | 54.58 |

--------------------------------------- **Relative Risk under H_0_** ---------------------------------------

Table S11: **Number of converged simulation runs** (out of 10,000) for the relative risk under the null hypothesis

|  | **Number of converged simulation runs (out of 10000), risk ratio, null hypothesis** | | | | |
| --- | --- | --- | --- | --- | --- |
| **Number of studies** | **BBST** | **GLFR** | **HKSJ** | **DSL** | **COLL** |
| 2 | 8115 | 9209 | 9999 | 9999 | 9999 |
| 3 | 8253 | 9501 | 9999 | 9999 | 9999 |
| 4 | 8234 | 9586 | 10000 | 10000 | 10000 |
| 5 | 8206 | 9536 | 10000 | 10000 | 10000 |
| 10 | 8252 | 9301 | 10000 | 10000 | 10000 |

Table S12: **(Absolute) bias** for the log relative risk under the null hypothesis

| **Number of studies** | **BBST** | **GLFR** | **HKSJ** | **DSL** | **COLL** |
| --- | --- | --- | --- | --- | --- |
|  | Median  Q1  Q3 | Median  Q1  Q3 | Median  Q1  Q3 | Median  Q1  Q3 | Median  Q1  Q3 |
| 2 | 0.0081  −0.3387  0.3448 | −0.0035  −0.2751  0.2486 | −0.0022  −0.2701  0.2573 | −0.0022  −0.2701  0.2573 | 0.0041  −0.2717  0.2963 |
| 3 | 0.0187  −0.2624  0.3180 | −0.0003  −0.2594  0.2478 | 0.0055  −0.2071  0.2315 | 0.0078  −0.2013  0.2357 | 0.0157  −0.2004  0.2946 |
| 4 | 0.0157  −0.2206  0.2659 | −0.0003  −0.2002  0.2159 | 0.0039  −0.1710  0.1992 | 0.0065  −0.1652  0.2028 | 0.0153  −0.1654  0.2549 |
| 5 | 0.0224  −0.1768  0.2413 | 0.0019  −0.1777  0.1999 | 0.0045  −0.1469  0.1776 | 0.0088  −0.1379  0.1832 | 0.0178  −0.1346  0.2383 |
| 10 | 0.0195  −0.1161  0.1771 | 0.0053  −0.1182  0.1466 | 0.0073  −0.0940  0.1350 | 0.0102  −0.0884  0.1402 | 0.0202  −0.0824  0.2045 |

Q1: 1st quartile; Q3: 3rd quartile

Table S13: **Coverage probability (%)** for the relative risk under the null hypothesis

| **Number of studies** | **BBST** | | **GLFR** | **HKSJ** | **DSL** | **COLL** |
| --- | --- | --- | --- | --- | --- | --- |
|  | K – 1 | 2K – 2 |  |  |  |  |
| 2 | 99.91 | 98.46 | 99.91 | 99.78 | 90.98 | 82.49 |
| 3 | 99.03 | 96.74 | 98.82 | 98.67 | 92.20 | 80.38 |
| 4 | 98.43 | 96.02 | 98.31 | 98.34 | 92.60 | 79.24 |
| 5 | 98.15 | 95.94 | 97.99 | 97.90 | 92.91 | 77.11 |
| 10 | 96.61 | 95.29 | 96.19 | 96.15 | 92.59 | 73.99 |

Table S14: **Length of 95 % confidence interval** for the log relative risk under the null hypothesis

| **Number of studies** | **BBST** | **BBST** | **GLFR** | **HKSJ** | **DSL** | **COLL** |
| --- | --- | --- | --- | --- | --- | --- |
|  | K – 1 | 2K – 2 |  |  |  |  |
|  | Median  Q1  Q3 | Median  Q1  Q3 | Median  Q1  Q3 | Median  Q1  Q3 | Median  Q1  Q3 | Median  Q1  Q3 |
| 2 | 12.9208  6.6518  25.1151 | 4.3753  2.2525  8.5046 | 12.1637  5.3423  27.4024 | 10.8288  4.6560  22.4518 | 1.9534  0.8424  3.8051 | 1.3238  0.6034  3.1102 |
| 3 | 3.6960  1.8946  6.6133 | 2.3850  1.2226  4.2675 | 3.5650  1.5802  7.4097 | 3.3899  1.4857  6.1244 | 1.5488  0.7015  2.9595 | 1.0343  0.4890  2.3615 |
| 4 | 2.3669  1.2378  4.0640 | 1.8199  0.9517  3.1247 | 2.2988  1.0269  4.5911 | 2.1671  0.9811  3.8777 | 1.3115  0.6026  2.3877 | 0.8413  0.4121  1.9469 |
| 5 | 1.8549  0.9867  3.1117 | 1.5406  0.8195  2.5844 | 1.7689  0.8294  3.4659 | 1.6793  0.7901  3.0400 | 1.1298  0.5398  2.0958 | 0.7182  0.3542  1.6605 |
| 10 | 1.0814  0.5841  1.7799 | 1.0043  0.5425  1.6530 | 1.0001  0.4835  1.8969 | 0.9464  0.4583  1.7220 | 0.7664  0.3815  1.4357 | 0.4730  0.2409  1.1080 |

Q1: 1st quartile; Q3: 3rd quartile

--------------------------------------- **Relative Risk under H_1_** ---------------------------------------

Table S15: **Number of converged simulation runs** (out of 10,000) for the relative risk under the alternative hypothesis

| **Number of studies** | **BBST** | **GLFR** | **HKSJ** | **DSL** | **COLL** |
| --- | --- | --- | --- | --- | --- |
| 2 | 8187 | 9011 | 9999 | 9999 | 9999 |
| 3 | 8259 | 9427 | 9998 | 9998 | 9998 |
| 4 | 8189 | 9577 | 9999 | 9999 | 9999 |
| 5 | 8193 | 9550 | 10000 | 10000 | 10000 |
| 10 | 8351 | 9504 | 10000 | 10000 | 10000 |

Table S16: **(Absolute) bias** of the log relative risk under the alternative hypothesis

|  | **Bias, log risk ratio, alternative hypothesis** | | | | |
| --- | --- | --- | --- | --- | --- |
| **Number of studies** | **BBST** | **GLFR** | **HKSJ** | **DSL** | **COLL** |
|  | Median  Q1  Q3 | Median  Q1  Q3 | Median  Q1  Q3 | Median  Q1  Q3 | Median  Q1  Q3 |
| 2 | 0.0343  −0.3818  0.4180 | 0.0277  −0.2528  0.3472 | 0.0285  −0.2572  0.3590 | 0.0285  −0.2572  0.3590 | 0.0174  −0.3031  0.3646 |
| 3 | 0.0299  −0.2875  0.3634 | 0.0074  −0.2593  0.2867 | 0.0354  −0.1775  0.3222 | 0.0394  −0.1713  0.3252 | 0.0207  −0.2295  0.3220 |
| 4 | 0.0286  −0.2380  0.3022 | 0.0029  −0.2215  0.2384 | 0.0329  −0.1436  0.2697 | 0.0363  −0.1346  0.2753 | 0.0196  −0.1837  0.2806 |
| 5 | 0.0267  −0.2037  0.2742 | 0.0057  −0.2056  0.2084 | 0.0337  −0.1143  0.2502 | 0.0387  −0.1074  0.2599 | 0.0222  −0.1565  0.2651 |
| 10 | 0.0242  −0.1255  0.2051 | 0.0017  −0.1347  0.1530 | 0.0339  −0.0679  0.2110 | 0.0378  −0.0625  0.2202 | 0.0237  −0.0973  0.2203 |

Q1: 1st quartile; Q3: 3rd quartile

Table S17: **Percentage bias (%)** for the log relative risk under the alternative hypothesis

| **Number of studies** | **BBST** | **GLFR** | **HKSJ** | **DSL** | **COLL** |
| --- | --- | --- | --- | --- | --- |
|  | Median  Q1  Q3 | Median  Q1  Q3 | Median  Q1  Q3 | Median  Q1  Q3 | Median  Q1  Q3 |
| 2 | −10.04  −136.18  137.99 | −10.30  −123.94  104.31 | −10.74  −123.49  100.56 | −10.74  −123.49  100.51 | −7.45  −134.79  119.17 |
| 3 | −8.51  −120.74  95.51 | −2.81  −104.32  98.26 | −14.37  −109.55  69.84 | −15.21  −111.85  66.78 | −8.49  −122.65  88.79 |
| 4 | −7.49  −99.77  80.77 | −1.09  −86.15  86.02 | −12.31  −90.58  59.37 | −13.58  −94.83  56.68 | −8.19  −105.91  75.29 |
| 5 | −8.09  −91.23  67.86 | −2.37  −75.28  77.97 | −13.05  −86.13  48.85 | −15.04  -88.72  45.05 | −9.40  −101.03  64.32 |
| 10 | −7.43  −61.79  40.85 | −0.68  −50.30  49.71 | −12.91  −63.84  28.39 | −14.49  −66.42  26.05 | −9.20  −77.93  37.94 |

Q1: 1st quartile; Q3: 3rd quartile

Table S18: **Coverage probability (%)** for the relative risk under the alternative hypothesis

| **Number of studies** | **BBST** | | **GLFR** | **HKSJ** | **DSL** | **COLL** |
| --- | --- | --- | --- | --- | --- | --- |
|  | K – 1 | 2K – 2 |  |  |  |  |
| 2 | 99.87 | 98.41 | 99.93 | 99.86 | 91.27 | 82.96 |
| 3 | 98.75 | 96.54 | 98.82 | 98.64 | 91.89 | 81.13 |
| 4 | 97.81 | 95.74 | 98.05 | 97.90 | 91.92 | 78.77 |
| 5 | 97.08 | 94.68 | 97.53 | 97.11 | 91.56 | 77.01 |
| 10 | 94.97 | 93.27 | 95.66 | 95.05 | 90.67 | 74.64 |

Table S19 **Length of 95 % confidence interval** for the log relative risk under the alternative hypothesis

|  | **Length of 95 % confidence interval, log risk ratio, alternative hypothesis** | | | | | |
| --- | --- | --- | --- | --- | --- | --- |
| **Number of studies** | **BBST** | | **GLFR** | **HKSJ** | **DSL** | **COLL** |
|  | K – 1 | 2K – 2 |  |  |  |  |
|  | Median  Q1  Q3 | Median  Q1  Q3 | Median  Q1  Q3 | Median  Q1  Q3 | Median  Q1  Q3 | Median  Q1  Q3 |
| 2 | 14.2708  7.3463  28.0049 | 4.8325  2.4876  9.4832 | 13.1289  6.0293  28.6662 | 12.0169  5.3527  23.8137 | 2.1915  0.9722  4.0681 | 1.4918  0.6875  3.4518 |
| 3 | 4.0477  2.1563  7.1441 | 2.6120  1.3914  4.6100 | 3.9702  1.8422  7.9479 | 3.7495  1.7421  6.4189 | 1.7212  0.8162  3.1057 | 1.1709  0.5573  2.6054 |
| 4 | 2.5917  1.3634  4.4362 | 1.9927  1.0483  3.4109 | 2.4704  1.1337  4,9483 | 2.3537  1.0844  4.1043 | 1.4116  0.6619  2.5519 | 0.9281  0.4469  2.1403 |
| 5 | 2.0265  1.0954  3.4358 | 1.6832  0.9098  2.8536 | 1.9585  0.9228  3.7900 | 1.8420  0.8845  3.2098 | 1.2564  0.6107  2.2394 | 0.8140  0.3973  1.8692 |
| 10 | 1.1762  0.6461  1.8947 | 1.0924  0.6000  1.7597 | 1.1267  0.5519  2.0412 | 1.0584  0.5217  1.7961 | 0.8646  0.4378  1.5010 | 0.5421  0.2772  1.2346 |

Q1: 1st quartile; Q3: 3rd quartile

Table S20: **Power (%)** for the relative risk under the alternative hypothesis

| **Number of studies** | **BBST** | | **GLFR** | **HKSJ** | **DSL** | **COLL** |
| --- | --- | --- | --- | --- | --- | --- |
|  | K – 1 | 2K – 2 |  |  |  |  |
| 2 | 0.06 | 2.81 | 0.02 | 0.20 | 16.80 | 27.81 |
| 3 | 3.55 | 10.74 | 3.62 | 4.10 | 19.53 | 34.06 |
| 4 | 9.73 | 16.88 | 9.72 | 9.70 | 22.80 | 39.09 |
| 5 | 14.13 | 20.48 | 14.43 | 13.74 | 25.93 | 42.99 |
| 10 | 30.16 | 33.36 | 31.24 | 28.35 | 35.50 | 54.48 |
